# Decoding health status transitions of over 200 000 patients with traumatic brain injury from preceding injury to the injury event

**DOI:** 10.1038/s41598-022-08782-0

**Published:** 2022-04-04

**Authors:** Tatyana Mollayeva, Andrew Tran, Vincy Chan, Angela Colantonio, Mitchell Sutton, Michael D. Escobar

**Affiliations:** 1 KITE-Toronto Rehabilitation Institute, University Health Network, Toronto, Canada; 2 Rehabilitation Sciences Institute, Temerty Faculty of Medicine, University of Toronto, Toronto, Canada; 3 Acquired Brain Injury Research Lab, University of Toronto, Toronto, Canada; 4 Dalla Lana School of Public Health, Health Sciences Building, University of Toronto, 155 College Street, 6th Floor, Toronto, ON M5T 3M7 Canada; 5 Institute of Health Policy, Management and Evaluation, University of Toronto, Toronto, Canada; 6 Toronto Western Hospital University Health Network, Toronto, Canada; 7 Global Brain Health Institute, Institute of Neuroscience, Trinity College, Dublin, Ireland

**Keywords:** Risk factors, Brain injuries, Epidemiology

## Abstract

For centuries, the study of traumatic brain injury (TBI) has been centred on historical observation and analyses of personal, social, and environmental processes, which have been examined separately. Today, computation implementation and vast patient data repositories can enable a concurrent analysis of personal, social, and environmental processes, providing insight into changes in health status transitions over time. We applied computational and data visualization techniques to categorize decade-long health records of 235,003 patients with TBI in Canada, from preceding injury to the injury event itself. Our results highlighted that health status transition patterns in TBI emerged along with the projection of comorbidity where many disorders, social and environmental adversities preceding injury are reflected in external causes of injury and injury severity. The strongest associations between health status preceding TBI and health status at the injury event were between multiple body system pathology and advanced age-related brain pathology networks. The interwoven aspects of health status on a time continuum can influence post-injury trajectories and should be considered in TBI risk analysis to improve prevention, diagnosis, and care.

## Introduction

Health status has been traditionally considered to encompass different dimensions and has been defined by the World Health Organization as "a state of complete physical, mental, and social wellbeing, and not merely the absence of disease or infirmity"^1^. A recent discussion has highlighted the limitations of this definition and suggested that health status is rather a dynamic process that reflects a person's change, which is consistent with the life course perspective within an environment, and that the past influences the present^2,3^.

Research on the dynamic component of health status in traumatic brain injury (TBI), which refers to structural and/or physiological disruption of brain function due to an external force^4^, is rapidly developing in various contexts^4–6^. Recently, the view of TBI has shifted from that of an injury event, to a condition with lifelong consequences for both morbidity and mortality^5,6^. Furthermore, it has been proposed that TBI may not be a cause but rather an effect of multiple disorders associated with risk of falls and challenging behaviours, including epilepsy and substance-related disorders, among others^5–8^. With this in mind, recent advances have focused on the preventability of TBI following a head injury event and adverse TBI consequences^6,9^, which may be related to a wide range of risks within health status preceding the injury event, as well as the risk of an injury event itself ^10,11^.

The depiction of TBI as a disease process rather than an event has enhanced the understanding of health transition following the TBI event; however, the health status transition from the time preceding injury to an injury itself remains unclear, and the challenge of considering how health statuses of individual patients unfold differently over time remains. Some health-related conditions are chronic in nature and require continuous management^13–15^ at the time a person sustains an injury; in these situations, the disorder (e.g., cardiovascular, metabolic, and neurologic disorders) is more likely to be captured in the injury surveillance data, as these are considered in TBI management and care^16^. Other health-related conditions are temporal in nature (e.g., sprains and strains, acute intoxication, abuse) and may not be noted in the injury event but may increase the probability of an injury as a result of falls, such as among those with poor balance or confusion, thus reflecting changes at both the physiological and psychosomatic levels^17,18^. To complicate matters, most patients discharged directly from the emergency department (ED) receive a concussion diagnosis with non-specific complaints of headaches, dizziness, balance issues, and sensitivity to noise and light^18–21^. While the Glasgow Coma Scale (GCS) score allows for a well-defined designation of TBI severity in more severe injury events that include a loss of consciousness and post-traumatic amnesia, this scale lacks sensitivity for milder injuries, such as concussion^22^. The GCS score is also inadequate to explain progressive symptom evolution from a relatively minor external physical force in patients who present with multiple disorders that require concurrent treatment at the time of the injury event^23,24^. Furthermore, disorders often coalesce with each other and with age-related factors and social adversities, which creates a vastly complex web of possible correlations to account for in the study of health status transition in TBI.

One method to increase confidence in the characterisation of health status transitions is to apply computational approaches to longitudinal health status data of patients with TBI both preceding and at the time of TBI diagnoses, and to compare these data with those of patients without TBI who are individually matched to TBI patients by sex, age, place of residence, and income level. This would allow health status to be studied in relation to the difference between the cohorts, and at two time points. In our recently published study^6^, we developed an algorithm to sequence thousands of diagnosis codes within the International Statistical Classification of Diseases and Related Health Problems, Tenth Revision (ICD-10) in 235,003 unique patients with TBI and the same number of patients without TBI, who visited ED or acute care hospitals over a decade. A total of 43 factors of health status from the five-years period preceding the TBI event that differentiated patients without a TBI were extracted and internally validated. Taking advantage of these 43 factors that describe the health status preceding TBI, here we first present a new analysis of data from the injury event, characterising health status of the same unique patients at the injury event. We then report associations between the factors of health status preceding TBI and that of an injury event, followed by portrayal of hierarchical clusters in data matrix of health status transitions, from time preceding injury to the injury event itself. We utilised the following steps in the analysis and validation process of the injury event phase and health status transition from the time of preceding injury to the TBI event phase: (1) determining the phase preceding TBI and the TBI event phase; (2) multiple testing to detect a set of definable health status patterns in TBI vs. non-TBI diagnoses; (3) factor analysis of health status patterns that are significantly related to TBI vs. non-TBI events; (4) a conditional logistic regression model and correlation matrix and hierarchical clustering using correlation-based distance to group health statuses at the TBI event; (5) health status transitions from the time preceding TBI^6^ to the TBI event, grouping all factors from each period into a single heatmap using agglomerative hierarchical clustering with interpretation of factors preceding and at the TBI event that are clustered together, to examine how many meaningful dimensions can be distinguished; and (6) internal validation of the results at each level analysis. Using this process, we confirmed that health status preceding injury is reflected in the injury event health status, and we provide evidence that health status preceding injury can explain the external cause of TBI and contribute to injury severity designation. These results provide a means to connect information on health status transitions in TBI and associated factors, from the time preceding injury to the injury event itself.

## Methods

### Population and health status data

We accessed the data from ICES^25^, which collects and stores health administrative data on publicly funded services provided to residents of Ontario, Canada, including information on acute care hospitalisations and ED visits. With nearly 14 million residents, Ontario is Canada's most populous province, comprising 43% of Canada's population^26^. Universal health care covers all medically necessary healthcare services at the point of care. The standardised discharge summary includes patient demographics and main and secondary diagnoses according to ICD-10 codes^27^. The ICD-10 codes consist of a combination of alphanumeric characters that characterise broad diagnosis categories. Each code is designed as an alphanumeric code and arranged hierarchically, with the code length ranging from 3 to 6 characters. The first three characters designate the category of the diagnosis, which is the same as the World Health Organisation's ICD-10 international standard for reporting diseases and health conditions^28^. The health service records data are linked deterministically at the individual level through a unique, encoded identifier based on name, sex, date of birth, and postal code. By applying unique de-identified health records, the health status trajectory of each patient can be tracked over time.

We used a previously established cohort of patients discharged between the fiscal years (defined as from April 1 to March 31) 2007/2008 and 2015/2016 from the ED (identified in the National Ambulatory Care Reporting System) and acute care (identified in the Discharge Abstract Database) with a diagnostic code for TBI (ICD-10 codes S02.0, S02.1, S02.3, S02.7, S02.8, S02.9, S04.0, S07.1, and S06)^6^; these patients comprised the TBI cohort in the present study. Patient demographics, main and secondary diagnoses, conditions, problems, or circumstances data^27^ were extracted for each individual patient. We selected a 10% random sample of patients discharged from the ED or acute care hospitals during the same study period for a reason other than TBI, and individually matched these to patients with TBI by sex, age, place of residence (urban vs. rural), and income quintile; these patients comprised the reference population^6^.

We followed previously published severity classifications^29,30^ to assign TBI injury severity. External causes of injury were determined using Centers for Disease Control (CDC) and Prevention major external cause of injury group codes, which were divided into falls, struck by/against an object, motor vehicle collision (MVC) and other causes^31^. We further identified assault-related TBI using ICD-10 codes specified by the CDC^31^. Sports-related injuries were identified based on the Association of Public Health Epidemiologists in Ontario (APHEO)^32^.

Step 1. **Determining the phase preceding TBI and the TBI event phase**
  The index date for patients with TBI was defined as their first occurrence of TBI over the study period, whereas, for the reference population, the index date was the midpoint of the ED or acute care visits. Data from 235,003 unique patients with TBI (and a same number of reference patients) were randomly split into training (50%; n = 117,689), validation (25%; n = 58,798), and testing (25%; n = 58,516) datasets. All analyses were completed and reported using the testing dataset, and the training and validation datasets were used for internal validation.
Step 2. **Multiple testing to detect a set of definable health status patterns in TBI vs. non-TBI diagnoses**
  *Health status preceding the injury event*
    We evaluated the health status preceding a TBI event reported by previous studies^6,33^. From all the possible ICD-10 codes classifying patients' main and secondary diagnoses, a previous data mining and validation study identified 43 factors that were significantly overrepresented in patients with TBI compared to reference patients (individually matched based on sex, age, place of residence, and income quintile) within the 5 years preceding their TBI event. For details, please see the study^6^.
  *Health status at the injury event*
    To identify health status at the TBI event and to gain insight into its observed correlations, we analyzed all ICD-10 codes depicted across the 10 and 25 diagnoses fields of the National Ambulatory Care Reporting System and the Discharge Abstract Database, respectively, for patients with TBI and reference patients at the index date. We converted all 2,600 codes into binary variables, except for provisional codes for research and temporary assignments, U98 and U99. The first three characters (one alphabetic and two numeric) of the ICD-10 comprised 2,600 distinct codes that defined specific diagnoses. These 2,600 binary ICD-10 code variables were tested for significant correlations with TBI diagnosis codes using a matched McNemar test with correction for multiple testing^34^. The Benjamini-Yekutieli method was applied to acquire a set of codes controlled at a False Discovery Rate (FDR) of 5%^35,36^. We identified ICD-10 codes that were associated with a TBI event, for which we then calculated odds ratios (ORs) to compare with the reference population (Supplementary File). To eliminate measurement artifacts, the procedure was first performed using the training dataset and then repeated using the validation dataset^37^. Only codes that were significant in both the training and validation sets were retained for further analysis.
Step 3. **Factor analysis of health status patterns that are significantly related to TBI vs. non-TBI events**
  To gain insight into the dimensionality structure of individual diagnosis codes, we performed factor analysis using principal components methods^38^. The optimal number of factors was determined by the breakpoint on the scree plot, eigenvalue, the greatest cumulative proportion of variance accounted for, and via a conditional logistic regression looped through all possible factors covering the largest area under the receiver operating characteristic curves^39^ (Supplementary Fig. 1, Supplementary Table 2).
Step 4. **Conditional logistic regression model and correlation matrix and hierarchical clustering**
  The conditional logistic regression model was built using binary factor-based scores^40^. Patients were assigned a score of one if they possessed any of the ICD-10 codes in the factor definition; otherwise, they were assigned a zero. These factor-based scores were used to calculate ORs and 95% confidence intervals from a conditional logistic regression model^40^ on the association between each factor and TBI, controlling for sex, age, rurality, and income in the testing dataset and then repeated in the training and validating datasets. To visualise the results of the factor analysis and conditional logistic regression model, a Pearson's correlation matrix was generated for all significant factors^41^, and hierarchical clustering was performed on similar group factors using correlation-based distance^42^ to identify groups of people with similar associative factors for TBI. To aid in the visualisation of clusters in the heatmap, clustering was performed using Ward (minimum variance) linkages^43^. The algorithms for these agglomerative clustering methods have been described elsewhere^43^.
Step 5. **Health status transitions from the time preceding TBI to the TBI event**
  To further expand our understanding of health status transitions from the time preceding TBI to the TBI event, we clustered all factors from each period into a single heatmap, where values of factors representing each time period were correlated. This was done using a Fisher transformation, which converted the correlations into "z-like statistics" ^44^. Next, factors preceding TBI were pooled into separate "injury severity" and "mechanisms of injury" event groupings.
Step 6. **Internal validation of the results**
  To determine the consistency of observed patterns, heatmaps were generated and compared between the training, validation, and testing dataset, with an FDR-corrected alpha set at 0.05. All correlations with adjusted p-values greater than 0.05 were set to 0 on the heatmaps, leaving only significant correlations with an FDR < 0.05.
  All analyses were conducted using SAS software (version 9.410, SAS Inc., Cary, NC) and R (version 3.4.1.11, R Foundation for Statistical Computing; www.r-project.org). Figures were created using R (ComplexHeatmap and Wordcloud, R Foundation for Statistical Computing; www.r-project.org).

### Ethical approval and informed consent

Approval: The study protocol was approved by the ethics committees at the clinical (University Health Network) and academic (University of Toronto) institutions. Accordance: All methods were carried out in accordance with the relevant guidelines and regulations. Informed consent: This research utilised de-identified health administrative data with no access to personal information. No humans were directly involved in this study.

## Results

Of the 58,516 patients in the testing dataset, 57% were male, and 43% were female. The most common TBI mechanisms were falls (n = 26,480 [45%]) and being struck by/against an object (n = 20,845 [36%]). Of all injuries, 25% were sports-related, and 10% were sustained in a MVC. Assaults accounted for 7% of TBIs. Injury severity was not established in 25,036 [43%] patients; most of these cases were recorded as concussion without a specified length of unconsciousness (ICD-10 code S06.0; Table 1 and Supplementary Table 3).

**Table 1:** Characteristics of patients with a first traumatic brain injury-related visit in the ED or acute care and matched reference patients.

Variables	Patients with TBI (N = 58,516)	Reference patients (N = 58,516)
**Sociodemographic characteristics**		
Sex, n (%)		
Male	33,379 (57)	33,379 (57)
Female	25,137 (43)	25,137 (43)
*Age at injury (years)*, mean (SD)	36.23 (25.33)	36.24 (25.32)
*Income quintile,* n (%)		
Q1 (lowest)	11,465 (20)	11,465 (20)
Q2	11,540 (20)	11,540 (20)
Q3	11,494 (20)	11,494 (20)
Q4	12,182 (20)	12,182 (20)
Q5 (highest)	11,835 (20)	11,835(20)
*Rural residence*	9,084 (16)	9,084 (16)
**TBI-related characteristics**		
*TBI main diagnosis,* n (%)	51,705 (88)	NA
*Injury severity,* n (%)		
Unspecified	25,036 (43)	NA
Mild	20,461 (35)	NA
Moderate	2,166 (4)	NA
Severe	10,853 (19)	NA
**Type of first healthcare entry, n (%)**		
Emergency Department	48,142 (82)	NA
Acute Care	4,147 (7)	NA
Emergency & Acute*	6,227 (11)	NA
External Cause and Context of Injury, n (%)**		
Sports injury	14,472 (25)	NA
Assault	4,359 (7)	NA
Falls	26,480 (45)	NA
Motor vehicle collisions	5,808 (10)	NA
Struck by/against	20,845 (36)	NA
Other	6,791 (12)	NA
Missing	166 (0)	NA

n/a = not applicable; TBI = traumatic brain injury; SD = standard deviation. Data given as mean (standard deviation) or n (%). *A patient had a transfer to either location on the same day. **A patient may have several designations (i.e., sports injury and struct by/against an object).

**Figure 1:**
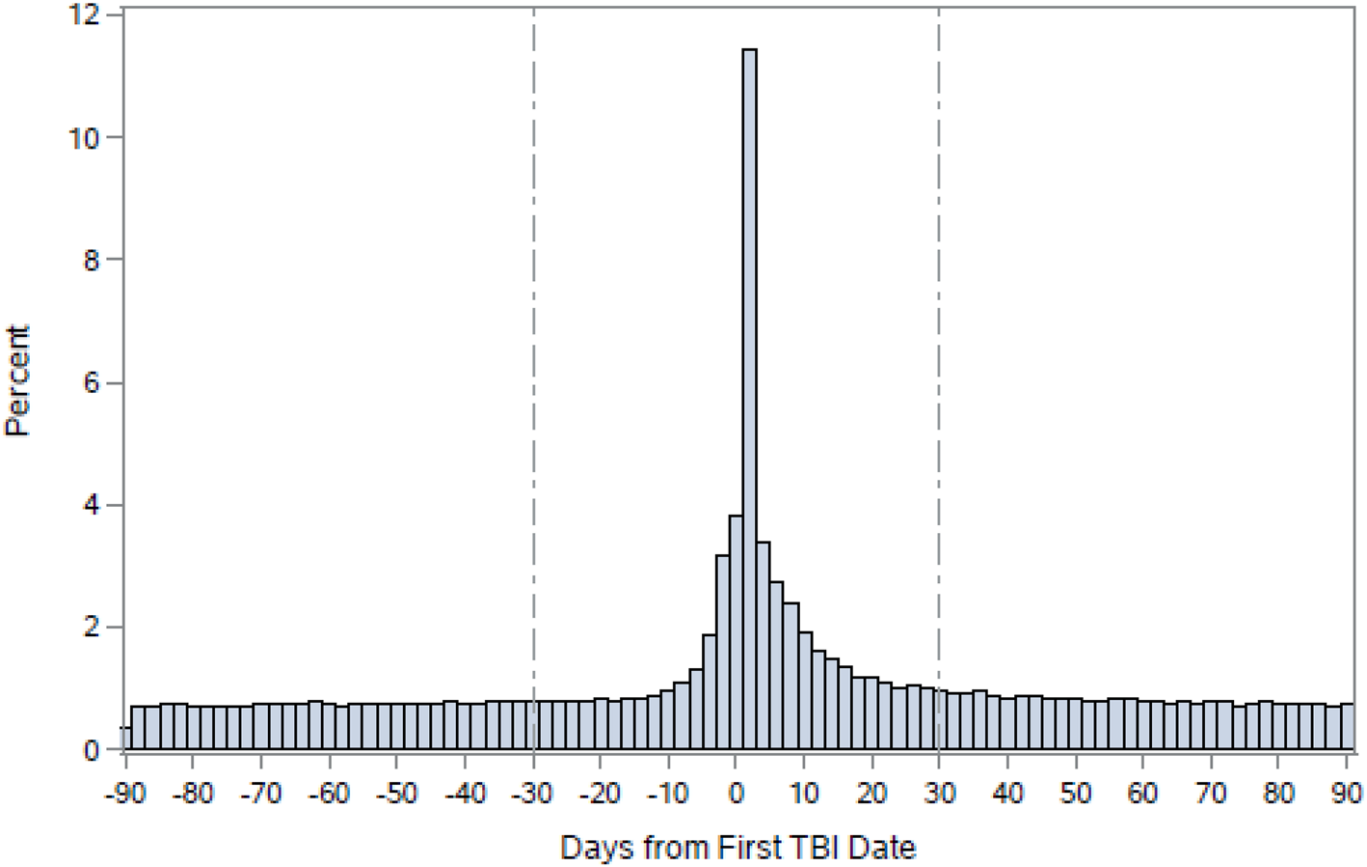
A number of hospital visits surrounding the TBI index date. Reprinted from Mollayeva, T. et al.^6^. The figure was originally published under a CC BY license (Creative Commons Attribution 4.0 International License).
Step 2. **Multiple testing to detect a set of definable health status patterns in TBI vs. non-TBI diagnoses**
  The matched McNemar tests were performed on the training dataset for 2,600 ICD-10 codes at the first three-character level, for patients with TBI and their matched reference patients, for significant associations with TBI diagnosis. The Benjamini-Yekutieli multiple testing, applied to acquire a set of codes controlled at a FDR of 5%, recognised 273 diagnoses codes that were significantly associated with TBI diagnosis (i.e., had an OR > 1). These codes were re-tested on the validation dataset, and 226 (83%) of them were internally validated (Supplementary Tables 3 and 4). Only codes that were significant in both the training and validation sets were retained for further factor analysis using the principal components method.
Step 3. **Factor analysis of health status patterns that are significantly related to TBI vs. non-TBI events**
  Factor analysis was applied to the training dataset. Of the 226 codes included in the analysis, 164 (73%) unique codes met the factor loading cut-off of 0.2 (Supplementary Fig. 2). For details on frequencies, ORs, and factor loadings of codes that met the factor analysis cut-off and codes that did not meet the cut-off, see Supplementary Tables 4 and 5, respectively. Using the breakpoints on the scree plots and the interpretability, 35 factors were selected. One factor (asphyxiation, suicide) had low frequencies in the reference population (< 6) and was excluded from further analyses. The remaining 34 factors were studied further. Figure 2 presents each factor by injury severity share.

Step 1. **Determining the phase preceding TBI and the TBI event phase**
  We found that ED and acute care visits prior to and following the TBI event (i.e., index date for TBI) followed a certain trend, whereby they appeared to plateau 30 days before and after the index date and remained largely unchanged after that (Fig. 1). Therefore, this 61-day period was defined as the TBI event window, whereas all ED and acute care visits within five years up to 30 days prior to a TBI event were considered to be the pre-injury phase. A similar procedure was performed for each patient in the reference population sample, with the exception that the midpoint of each patient's ED and acute care visits was selected as an index date.

**Figure 2:**
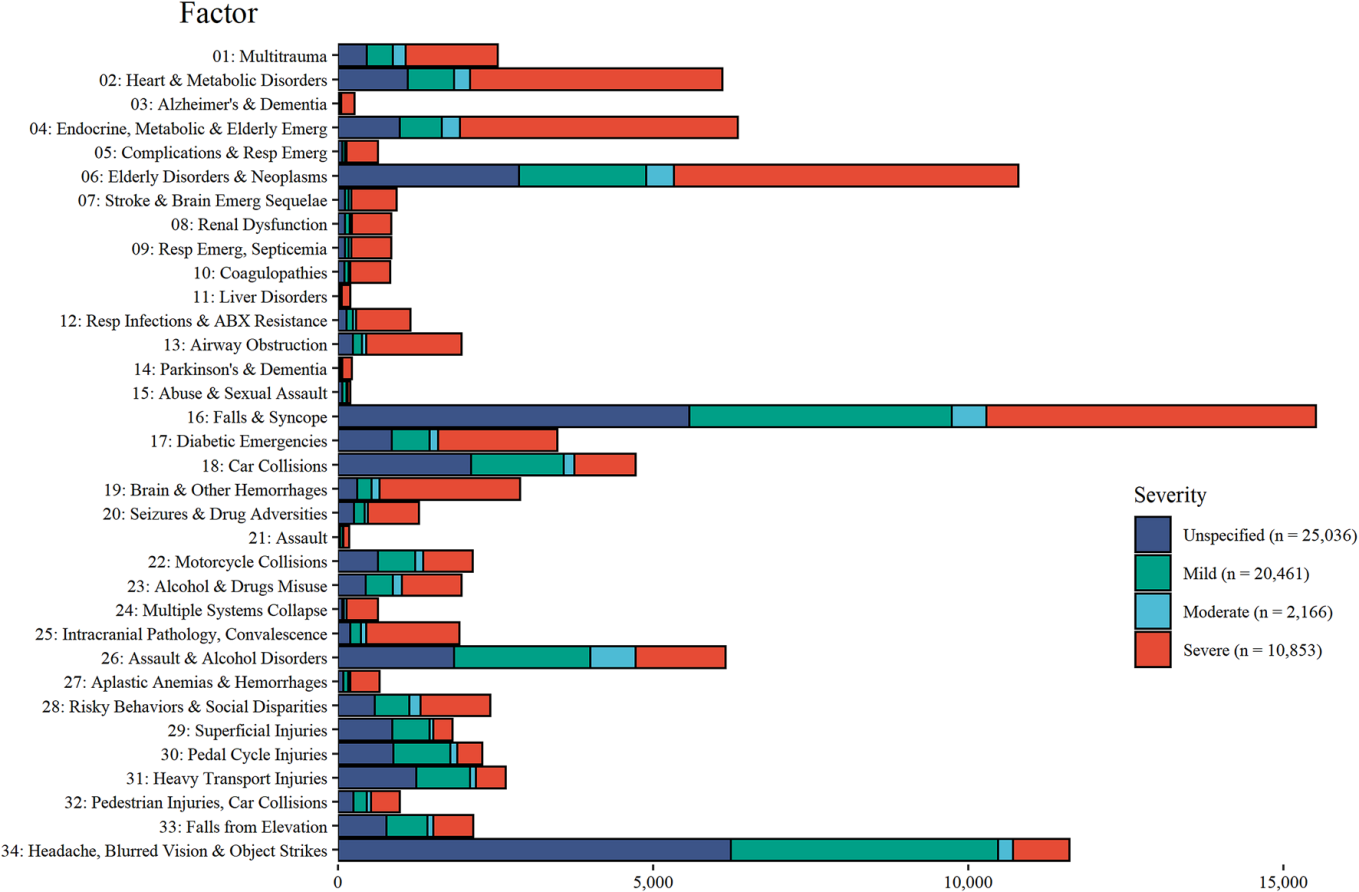
Health status factors at the TBI event phase by injury severity in patients with TBI in Ontario, Canada 2002–2016. The total number of each health status factor in TBI event across the sample set (n = 58,516). Data are shown for each injury severity, coloured by mild, moderate, severe, and unspecified. Abbreviations: ABX = antibiotics; Emerg= emergency; Resp= respiratory
  Table 2 presents the descriptions, frequencies, ORs, and ICD-10 codes included for each of the 34 factors. Supplementary Table 4 presents factor loadings and detailed descriptions of each factor.

**Table 2:** Factor analyses with ICD-10-CA codes, disease category and effect size (OR and 95% CI).

Factor number	Short description	Category	ICD-10 codes	Frequency in cohorts		OR [95% CI]
				TBI	Ref	
Factor 1	Multitrauma	Emergency Medicine	S36, S27, S37, T06, T79, S42, S26, S72	2535	749	3.49 [3.21–3.79]
Factor 2	Heart & Metabolic Disorders	Cardiology	E78, I10, I48, Z95, I50, Z92, E03, E11	6095	3,787	1.97 [1.88–2.07]
Factor 3	Alzheimer’s & Dementia	Neurology	F00, G30	267	79	3.41 [2.65–4.39]
Factor 4	Endocrine, Metabolic & Elderly Disorder	Emergency Medicine/Geriatrics	I10, E87, E83, E22, F05, Z75, N17, F06, B96, I95, R41	6338	2,873	2.85 [2.70–3.00]
Factor 5	Complications & Respiratory Disorders	Emergency Medicine	J95, Y84, J15	638	204	3.14 [2.68–3.68]
Factor 6	Elderly Disorders & Neoplasms	Geriatrics	S72, F05, Z75, R41, F03, Z51, R29, W05, R26, W19, C79, Z74, W06	10,793	2,275	6.92 [6.54–7.31]
Factor 7	Stroke & Brain Emerg Sequelae	Neurology/Physical Medicine and Rehabilitation	G81, R47, I63, I69, R13	929	322	2.96 [2.61–3.37]
Factor 8	Renal Dysfunction	Nephrology	N17, Z99, N18, N08	845	439	2.00 [1.77–2.25]
Factor 9	Respiratory Emergencies, Septicaemia	Emergency Medicine	N17, J17, A41, J96	844	378	2.32 [2.05–2.63]
Factor 10	Coagulopathies	Haematology	Z92, Y44, D68	826	183	4.65 [3.95–5.48]
Factor 11	Liver Disorders	Gastroenterology	K70, R18, K72, B18	198	71	2.79 [2.13–3.66]
Factor 12	Respiratory Infections & ABX Resistance	Infectious Diseases	B96, B95, U82, A49, L89	1147	570	2.09 [1.88–2.31]
Factor 13	Airway Obstruction	Emergency Medicine	Z51, R13, J96, L89, J69, W80	1956	771	2.70 [2.48–2.95]
Factor 14	Parkinson’s & Dementia	Neurology	F02, G20, G31	221	50	4.42 [3.25–6.01]
Factor 15	Abuse & Sexual Assault	Family medicine/ Psychiatry/Trauma	T74, Y07, Y05, Y06	196	15	13.07 [7.73–22.09]
Factor 16	Syncope & Falls	Emergency Medicine/Mechanism	I95, W19, R55, W18, W01	15,516	3,106	6.79 [6.49–7.11]
Factor 17	Diabetic Emergencies	Emergency Medicine	E11, N08, E14, R73, G63	3479	2,426	1.54 [1.45–1.63]
Factor 18	Car Collision	External cause of injury	V43, V49, V48, V89, V47, T14, Z04, V58	4723	711	7.14 [6.58–7.75]
Factor 19	Brain & Other Haemorrhages	Neurology	Z51, C79, I61, I60, G91, I62, G06, I67, Z54	2,887	744	4.24 [3.90–4.62]
Factor 20	Seizures & Drug Adversities	Neurology/Pharmacology Emergencies	Y46, G40, T42, R56, G41, R27	184	288	4.56 [4.00–5.19]
Factor 21	Assault	External cause of injury	X99, S21, S11, S15	178	33	5.39 [3.72–7.82]
Factor 22	Motorcycle Accidents	External cause of injury	S42, V28, V29, V27, V23, V86, V22	2133	674	3.27 [3.00–3.58]
Factor 23	Alcohol & Drugs Misuse	Psychiatry	Y90, R78, F10	1,960	435	4.66 [4.19–5.18]
Factor 24	Multiple Systems Collapse	Emergency Medicine /Neurosurgery	G93, Z52, D65, E23, I46	629	76	8.28 [6.53–10.51]
Factor 25	Intracranial Pathology, Convalescence	Emergency Medicine/Physical Medicine and Rehabilitation	S72, Z51, C79, I62, Z54, Z50	1925	363	5.75 [5.11–6.46]
Factor 26	Assault & Alcohol Disorders	Psychiatry/ Population and Community Health/ Mechanism	F10, Y04, Y09, Y00, H05, H11, H53, Y08	6151	762	9.30 [8.58–10.09]
Factor 27	Aplastic Anaemias & Haemorrhages	Haematology/Neurology	D46, D61, D69, D64	659	455	1.46 [1.29–1.65]
Factor 28	Risky Behaviours & Social Disparities	Family Medicine/ Psychiatry/Population and Community Health	B18, F10, F14, F11, Z59, Z91, Z72, Z21	2417	694	3.64 [3.34–3.97]
Factor 29	Superficial Injuries	Emergency Medicine	S30, S40, S70, S20, S10, S39	1,813	894	2.06 [1.90–2.23]
Factor 30	Pedal Cycle Injuries	External cause of injury	V18, V19, T00, V13, V17	2292	514	4.64 [4.21–5.13]
Factor 31	Heavy Transport Injuries	External cause of injury	V43, V44, V54, S19	2661	298	9.53 [8.42–10.79]
Factor 32	Pedestrian Injuries, Car Crashes	External cause of injury	S15, V03, V09, T08, T02, I72	982	92	10.89 [8.78–13.52]
Factor 33	Falls from Elevation	External cause of injury	W17, W13, W11, W12	2147	296	7.59 [6.70–8.59]
Factor 34	Headache, Blurred Vision & Object Strikes	Neurology/Mechanism	R51, F07, G44, W22, Z02, W20	11,604	2,079	6.80 [6.45–7.16]

**Figure 3:**
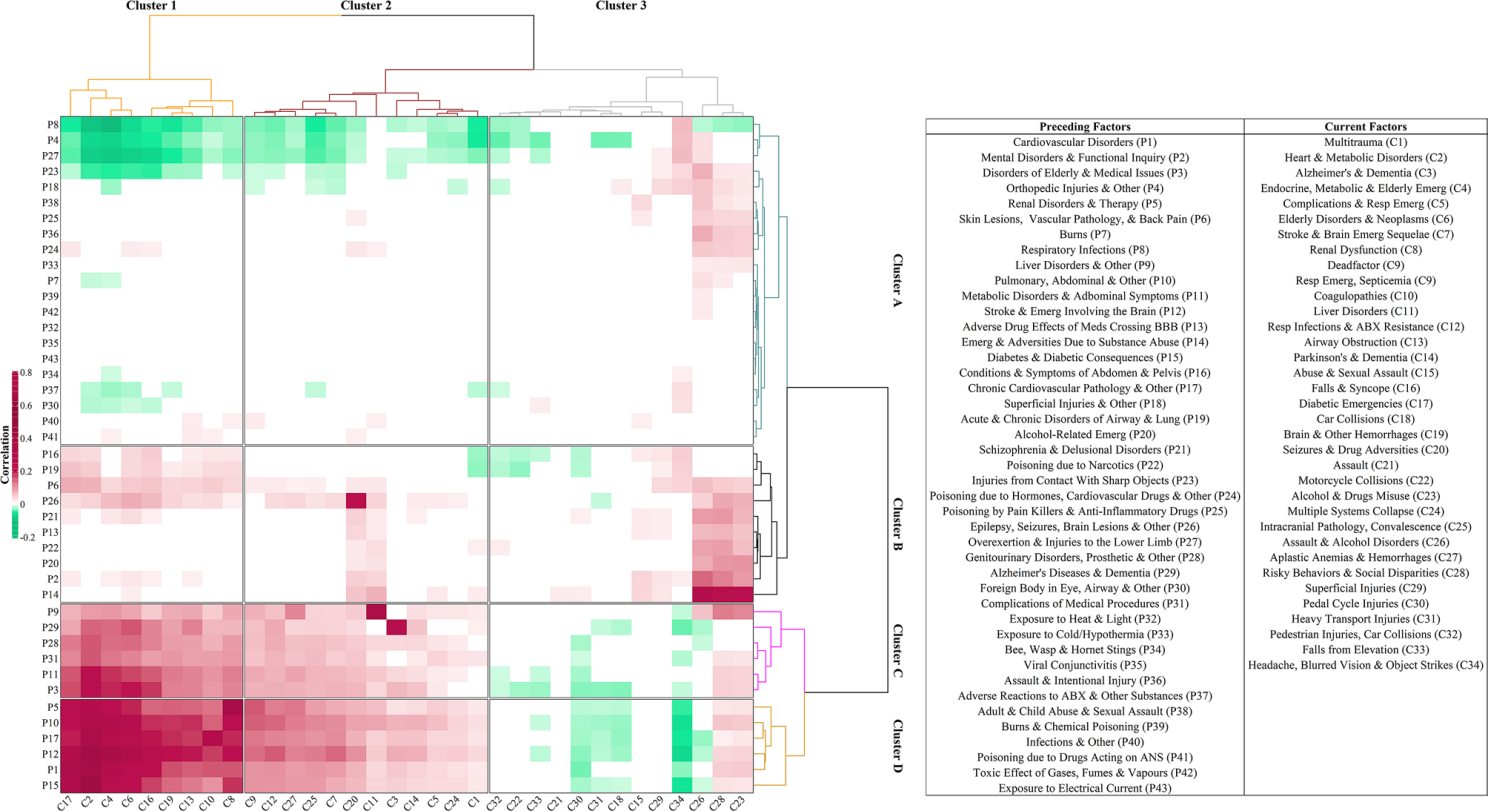
Cluster analysis and heatmap across 43 factors preceding TBI (y-axis) and 34 factors at the TBI event (x-axis). On the left y-axis, preceding injury clusters (Clusters A-D) are annotated for reference with the text. On the upper x-axis, TBI event clusters (Clusters 1–3) are likewise annotated. Annotations are presented as guidelines and are not definitive. Only internally validated factors in the testing and validation datasets are presented. In the heatmap, each colour represents a set of binned ranks in the heatmap, with green colours representing negative correlations and magenta colours representing positive correlations, adjusted for FDR. White fields represent non-significant correlations after adjustment for FDR.
Step 5. **Health status transitions from the time preceding TBI to the TBI event**
  The strongest positive correlations between health status preceding TBI and health status at the injury event were between Cluster D and Cluster 1 (i.e., multiple body system pathology) and Cluster C and Cluster 2 (i.e., advanced age-related brain pathology). The multiple body system pathology was composed of endocrine system pathology, i.e., diabetes and diabetic emergencies (Factors P15 and Factor C17), cardiovascular system pathology (Factor P1 and Factor C2), alterations in renal and urinary tract function (Factor P5 and Factor C8 ), and brain haemorrhages and stroke (Factor P12 and Factor C19). The advanced age-related brain pathology consisted of liver disorders (Factor C11 and Factor P9), Alzheimer's disease and dementia (Factor P29 and Factor C3), and aplastic anaemias and haemorrhages and liver disorders (Factor P9 and Factor C27), among other advanced neurological sequelae.
  Cluster B preceding TBI (i.e., poisons, drug overdose, social adversity) was strongly associated with multiple pathologies at the injury event (Clusters 1–3), including seizures and drug adversities (Factor P26 and Factor C20) and illnesses due to poisons and drug overdose (Factor P9 and Factor C23; Factor P13 and Factor C26). Weaker correlations were observed between multiple body system pathology (Cluster 1) and advanced age-related brain pathology at the injury event and Cluster A preceding injury (i.e., young age-related concerns), assault and intentional injury (Factors P28 and P36 and Factor C26), overexertion and superficial injuries, exposure to environmental adversities (i.e., burns, cold/hypothermia, exposure to heat/light), and lifestyle and adverse drug effect preceding injury (Factors P18, P7, P33, and Factors C23 and C28).
  *Health status preceding injury associates with injury severity and external causes of injury*
    Many of the health status factors preceding TBI showed a significant association with TBI severity, thereby likely contributing to the GCS score at the time of injury. For example, severe TBI and age at injury were characterized by a link to Clusters C and D preceding injury (i.e., multiple body system pathology and advanced age-related brain pathology, Fig. 4), which comprised metabolic disorders (Factors P9, P11, P15), neurological disorders (Factor P12), cardiovascular pathology (Factors P1 and P17), Alzheimer's disease and dementia (Factor P29), and disorders of older people (Factor P3). In contrast, respiratory infections, musculoskeletal (MSK) injuries, and overexertion in Cluster A (i.e., young age-related concerns, Factors P8, P4, and P27) preceding TBI were negatively correlated with severe TBI. A reverse health status association was observed for the mild and unspecified TBI severity, whereas moderate TBI severity showed positive correlations with the cluster of disorders associated with poisoning due to narcotics, substance abuse, and liver pathology preceding injury (Factors P22, P14, and P9). This clustering analysis was performed separately for the training, validation, and testing datasets, and consistent patterns in the clustering of health status factors with injury severity were observed across each dataset.

ABX = antibiotics; CI = confidence interval; OR = odds ratio; TBI = traumatic brain injury; SD = standard deviation. Frequencies given as numbers.
Step 4. **Conditional logistic regression model, correlation matrix, and hierarchical clustering**
  Heatmaps of factors preceding TBI and factors of the TBI event are presented in Fig. 3.

**Figure 4:**
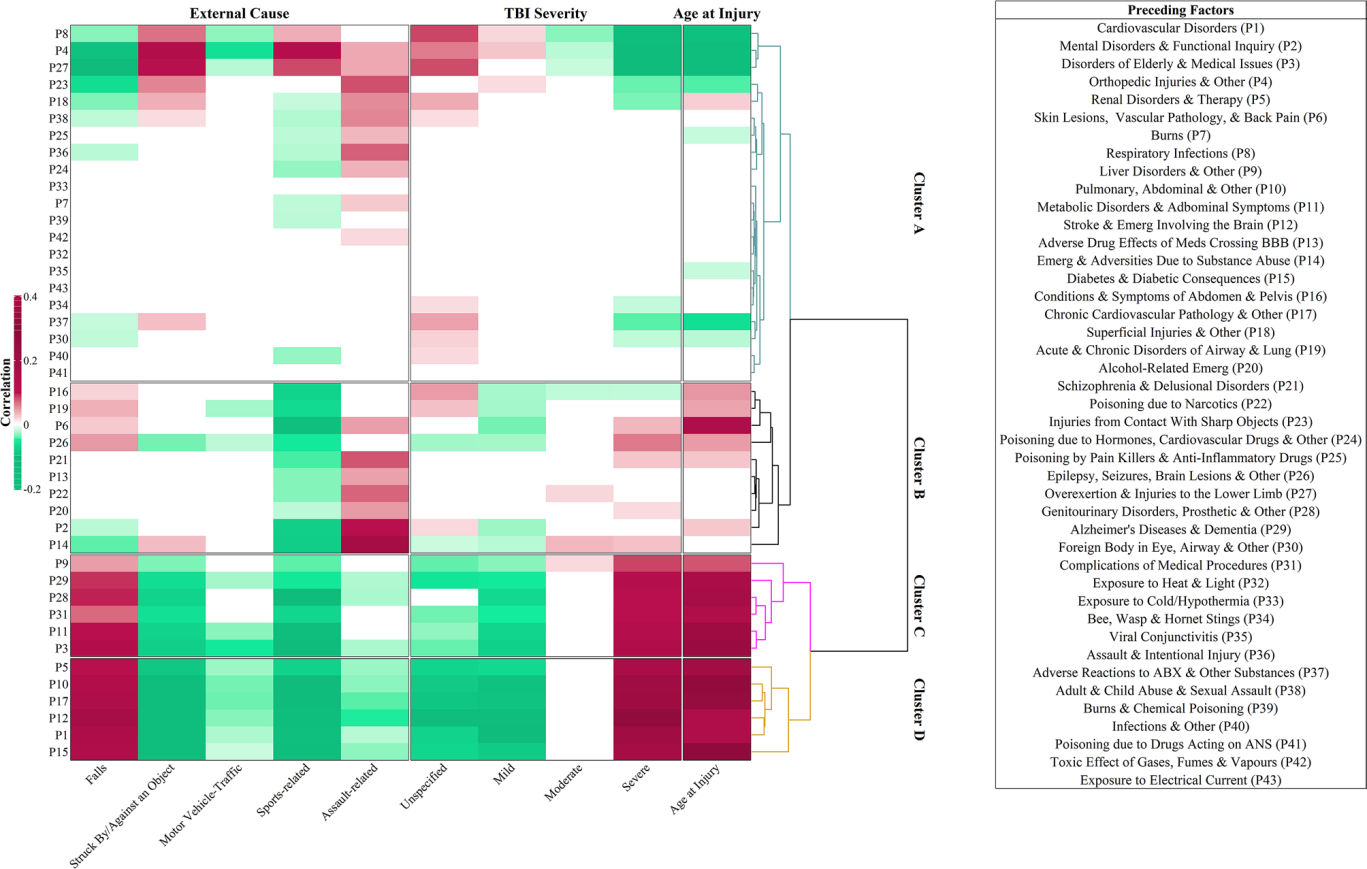
Cluster analysis and heatmap across 43 factors preceding TBI (y-axis) and mechanism, context of injury and TBI severity (x-axis). On the right y-axis, sample-based clusters are observed. Annotations are presented as guidelines and are not definitive. Only factors that were internally validated in the testing and validation datasets are represented. In the heatmap, each colour represents a set of binned ranks in the heatmap, with green colours representing negative correlations and magenta colours representing positive correlations, adjusted for FDR. White fields represent non-significant correlations after adjustment for FDR.
    External causes of injury were also distinguished by combined clusters of health status factors preceding TBI. Falls were characterised by a strong positive correlation with Clusters C and D (i.e., multiple body system pathology and advanced age-related brain pathology), mimicking the clusters associated with severe TBI, and a strong negative correlation with Cluster A (i.e., young age-related concerns), mimicking the clusters associated with mild and unspecified TBI severity. In contrast, struck by/against an object showed a strong positive correlation with Cluster A and negative correlations with Clusters C and D.
    A few patterns of weak negative correlations were observed between health status preceding TBI and MVC as an external cause of injury, whereas sport-related and assault-related causes of injury showed distinct positive correlations with health status preceding TBI. Meaningful observations included clustering of respiratory infections preceding injury with orthopaedic injuries and overexertion (Cluster A, Factors P8 with P4 and P27) in sport-related injury, and preceding injury poisoning by drugs and other substances, assault and abuse, and injuries from contact with sharp objects (Cluster B, Factors P23, P24, P36, and P38) with assault-related TBI.
Step 6. **Internal validation**
  To determine the consistency of the observed patterns, clustering analysis and heatmaps were generated and compared between the training, validation, and testing datasets. All reported results were confirmed in the training and validation datasets, and clusters and heatmaps were shown to be robust (Supplementary Figs. 2 and 3).

## Discussion

In this paper, we described a method for aligning health status transition in TBI, a disorder of significant public health concern and a major cause of disability worldwide^4,45^. The methods presented here describe a non-hypothesis-driven approach for detecting health status at injury events and combining them with health status results preceding the injury. This approach offers an explanation for the challenges associated with injury diagnosis, classification, and surveillance, which can be confounded by population health heterogeneity and epigenetic ambiguity^46^. With these challenges in mind, we conducted an impartial and interpretable assessment of health status transitions in TBI accounting for 2,600 individual diagnoses encoded using the ICD-10^3^ in a retrospective cohort of people of all ages, biological sex, socioeconomic standing, and place of living who had universally-funded access to healthcare. We internally validated our results and found them to be robust. We believe that the presented method to study health status transitions in TBI will spur the development of additional methods and prove useful for future analyses on health status transition after the injury event. Application of a health status transitions perspective to contextual injury event, and recognition that health status preceding injury makes a person more or less susceptible to TBI due to specific external cause of injury and developing a more or less severe TBI, entails new approaches to injury taxonomy, treatment and rehabilitation, and predictive classifications. This is important to avoid transition bias that can arise when people are prognostically different at the injury event phase because of their health status preceding injury. The results encourage dialogue among researchers, clinicians, and policymakers on health status transition perspective in TBI and other complex disorders and injuries.

Our results demonstrate that the transitions in health status from the time preceding injury and the injury event are depicted in the patterns of associations, external cause of injury and injury severity. We observed both hidden transitions, when the person's exposures preceding injury were not a constituent of the health status captured in the TBI event (i.e., exposures to gases and fumes, electrical currents, sharp objects, machinery, as shown by white fields in Figs. 3 and 4), as well as observed transitions, when the health status preceding injury was contained in the assessment of the injury event. Such transitions include cardiovascular, endocrine, metabolic, and neurological disorders, and disorders of the elderly, that were not resolute with time, and which were significantly reflected in the TBI event's external cause of injury and injury severity (magenta and green fields in Figs. 3 and 4). Together, these results suggest that patterns in the health status transition of patients with TBI emerge along the course of their comorbidity, which is consistent with previous reports^45,47–51^.

Our results suggest that many disorders preceding injury are reflected in external causes of injury and injury severity. Disorders clustering within the same external cause of injury and injury severity, as highlighted here, illuminate TBI as an event that is constructed within the context of health and social statuses, both formative and reflective^6,52^. For example, we observed that clusters composed of cardiovascular and metabolic disorders, stroke, dementia, and disorders of the elderly preceding TBI were strongly associated with falls and severe TBI. While the above disorders, individually, have long been known to be implicated in the risk of falls^53–55^, we demonstrated their formative links, both with other disorders and with TBI diagnosis. The association of Clusters C and D with TBI severity found here has significant diagnostic relevance. In this regard, both the depth and duration of coma following the injury event have been considered as an injury severity indicator using the GCS score^56^. While it has been previously suggested that GCS scores can be affected by intoxication, hypoxia, and hypotension, among other things^57–59^, the health status and age of patients presenting with these signs are not currently accounted for when determining injury severity. This has both clinical and policy implications, as there is a continuing debate over use of the GCS score in trauma patients of all ages, including preverbal children, to determine the time to extubation^60^, sedate^60^, and withdraw life support^61^, as well as intensive care stay duration^62^, rehabilitation^63^, discharge destination^64^, resource utilisation^65^, and litigation^66^.

A large number of patients with TBI in our sample (43%) did not have an established injury severity; most of these events were coded as concussions without a specified length of unconsciousness (S06.0 codes). The links between MSK illnesses preceding injury event and unspecified TBI severity sustained in a sports-related context have been described previously^6^; however their clustering on young age-related disorders (i.e., respiratory infections and adverse reaction to antibiotics (Factors P8 and P37), overexertion (Factor P27), adult and child abuse and assault (Factor P38), and foreign body in eye or airway (Factor P30) preceding injury) are novel findings. They may highlight the limitations of establishing level of responsiveness according to three aspects – eye-opening, motor, and verbal responses – in compromised person-environment and healthcare interactions, as well as a greater probability of an unwitnessed injury event in such interactions.

Finally, our results provide a basis for using pre-injury health status as an integral part of precision medicine and injury surveillance. We found clusters of factors associated with severe injury and those with mild injury severity and concussion. Factors clustering on moderate-to-severe TBI are composed of system-level disorders, poisons, and drug overdose, and those associated with mild TBI and unspecified injury severity (i.e., concussion codes) are composed of MSK-related injuries and respiratory illnesses. The external cause of injury, especially falls and struck by/against an object, nearly clustered in accordance to the severity, with falls linked to patients with system-level and neurological disorders and severe TBI, and being struck by/against an object linked to superficial injuries, overexertion, orthopaedic injuries, and mild TBI and concussions^67,68^. Notably, MVCs showed very few associations, all negative, the most significant of which, across clusters, were orthopaedic injuries, seizure disorders, and disorders of older people; these conditions linked to obstacles to, or lack of authorisation to operate a machinery^69–71^.

As presented in this research, we developed a feasible method to work with big data and complex clinical and public health topics in TBI simultaneously, which can be applied to other complex disorders and injuries. We have shown that it is possible to convert thousands of diagnoses encoded within the ICD-10 structure into hundreds of TBI-related diagnoses, and then further reduce these diagnoses into few dozens of factors that collectively explain the TBI event's significantly shared variance with factors preceding TBI. We created a procedure for visualised cluster analysis and heatmaps to accurately trace health status transitions and to detect and localise the clusters associated with the transitions. Encouraged by meaningful observations, we adapted and extended the analyses to external causes of injury and injury severity, and provided evidence that health status preceding an injury event is reflected in the injury event, as TBI-event health status and factors were implicated in the cause of injury and injury severity designation. Depending on the cluster and its formation, we anticipate that our analyses could offer new important information on injury severity in the case of falls and being struck by/against an object, and assault-related injury surveillance.

Despite the scientific and technological advances captured in this work, there are still questions to be addressed in future research. The data-driven approach we developed and the results are based on ED and acute care hospital records; there are still persons with TBI who may choose to be treated at a primary care facility within the healthcare system, which could be an additional source for coding injury and morbidity data. Primary care data should be explored in the future, given that hospital data tends to be efficient and does not always strive for completeness^27,28^. This is important for assault-related injury surveillance, as the ICD codes do not capture the victim/perpetrator relationship, e.g.,  TBIs due to intimate-partner violence. In Ontario, a code is mandatory only when the condition or circumstance exists at the patient's visit and is significant to the patient's treatment or care^28^. In the future, we plan further investigation and external validation of health status preceding injury, especially for circumstances not reflected in the TBI event window, but which were implicated in the external cause and severity of the injury, linking them to recovery and functional trajectory. In addition, we used the first three characters in the ICD-10, which designate the category of the diagnosis at the time preceding an injury event^72^. By using the whole sequence of codes instead of the first three characters, more data can be preserved for model testing, training, and validation; however, this necessitates a higher computing power to run the analyses^72^. Finally, despite the reliability/validity of ICES data on ED and acute care visits^73,74^, there may remain uncovered variation between Ontarians due to differences in access to care and help-seeking behaviours. This may be especially true for certain external causes of TBI, for example, assault related TBIs sustained in rural settings^75^. In an effort to mitigate this issue, we internally validated the results that emerged in the testing dataset using the training and validation datasets and detailed these results in the supplementary material of the manuscript. Nonetheless, future research would require ensuring generalizability by externally validating the described health status transitions using data from a patient population across Canada.

### Implications for prevention

Primary prevention seeks to circumvent injury before it occurs by protecting persons and vulnerable groups among the population^76^. Secondary prevention involves early recognition and targeting conditions that have already produced pathological change^77^, to stop the adverse injury course. Tertiary and quaternary prevention involves treatment directed to prevent long-term complications and minimize disability^78,79^. This research focused on time preceding injury and injury event, shown diagrammatically in Fig. 4, and, therefore, allowing the discussion of primary and secondary prevention initiatives.

Advanced age-related brain pathology (Cluster D) and disorders associated with poisoning due to narcotics, substance abuse and liver pathology (Cluster B) preceding TBI could be targeted in primary prevention. These relevant clusters preceding injury were associated with severe TBI, multiple pathologies, and other neurological sequelae at the time of injury. Interventions focusing on balance, posture, and moving equipoise training in the elderly has shown to be accompanied by a decline in falls^80–82^. Aside from targeting postural instability due to age-related motor impairments, advanced age-related brain pathologies highlighted in this work have been reported to challenge the risk–benefit ratio balance of treatment options with links to falls^83^. There has been a recent call to utilise a minimally disruptive approach when deciding on pharmacological management of conditions of the elderly to reduce the likelihood of drug interactions and falls prevention^84^.

Raising awareness about the links between poisoning due to narcotics, substance abuse, and liver pathology (Cluster B) preceding injury and assault related TBI is important^85^. While the ability of healthcare providers to prevent or modify such behaviors has not been proven, it might be possible to direct medical effort to the prevention of alcohol and drug-associated problems and, by that, prevent injury, violence, and medical complications of drug abuse^86^. Early detection and interventions that have proven effective for addictions include brief counselling, referral to ambulatory and inpatient treatment programs, community organizations, and appropriate medication use for substance use withdrawal^87–89^. Likewise, screening for exposure to relationship violence (i.e., adult and child abuse and sexual assault) and developing long-term plans and referrals to appropriate community and governmental agencies may prevent assault related TBIs^90^.

Ideas for secondary prevention strategies emerged from the results of this research include attention to interventions directed on the risk associated with the loss of patient autonomy in severe TBI cases, strongly linked to multiple body system pathology both preceding and at the time of injury (Cluster C and Cluster 1). Experimental therapies that inhibit the release of excitotoxins that play an important role in secondary injury to attenuate cellular oxidative and metabolic stress might prove to be effective^91^. Likewise, because of the substantial risks of repeated TBIs and adverse TBI outcomes from unaddressed narcotics, substance abuse and liver pathology (Cluster B), healthcare adoption of a broad construction of health status transition, considering family and social environments of their patients, is key^92^. Routinely eliciting information about the home, work, and neighbourhood exposures, and documenting family and social circumstances^93^ can help direct secondary prevention interventions and recommendations, as individual patients' situations will dictate feasible targets to which primary care providers should be alerted, when intended to ameliorate the course of TBI.

In summary, advances in data-driven analysis reveal a remarkable extent of meaningful associations in health status in the time preceding and following a TBI event that direct ideas for primary and secondary prevention. Possible extensions to this line of research would involve detecting health status transitions from the event to post-injury phase that could support tertiary and quaternary prevention, with compelling injury surveillance and public health ramifications.

### Clinical implications

The study results highlight clinical implications of health status transition in TBI, which necessitate integration of primary and secondary preventive practices into the care of individual patients. Despite challenges associated with limited reimbursement and time^94^, skepticism about patients' commitment to change^95^, and conflicting professional recommendations^96,97^, preventive practices fall under direct clinical provision of health promoting strategies and prophylactic treatment^98^.

## Supplementary Information

Supplementary Information.

## Data Availability

ICES is an independent, non-profit research institute funded by an annual grant from the Ontario Ministry of Health and Long-Term Care (MOHLTC). As a prescribed entity under Ontario's privacy legislation, ICES is authorized to collect and use health care data for the purposes of health system analysis, evaluation and decision support. Secure access to these data is governed by policies and procedures that are approved by the Information and Privacy Commissioner of Ontario. The dataset from this study is held securely in coded form at the Institute for Clinical Evaluative Sciences (ICES). While data sharing agreements prohibit ICES from making the dataset publicly available, access may be granted to those who meet pre-specified criteria for confidential access, available at www.ices.on.ca/DAS. The full dataset creation plan and underlying analytic code are available from the authors upon request, understanding that the computer programs may rely upon coding templates or macros that are unique to ICES and are therefore either inaccessible or may require modification.

## Glossary of terms

Benjamini-Yekutieli procedure: A statistical procedure that controls the false discovery rate under arbitrary dependence assumptions.
Cluster: A statistical classification technique in which a network of objects or points with similar characteristics are grouped in clusters
Cluster analysis: A statistical classification technique for exploratory data analysis in which a set of objects with similar characteristics are grouped in clusters; in this work, the technique was used for grouping similar kinds of factors into respective categories
Cluster heatmap: A visualization technique used to reveal hierarchical clusters in data matrix by drawing a rectangular grid corresponding to rows and columns and coloring the cells by their values in the data matrix; this technique has high data density and reveals clusters better than unordered heatmaps alone
Discharge Abstract Database: Captures administrative, clinical, and demographic information on hospital discharges (including deaths, sign-outs, and transfers); data is received directly from acute care facilities or their respective health/regional authority or ministry/department of health
Factor: A set of observed variables that have similar response patterns; they are associated with a hidden variable (called a confounding variable) that is not directly measured
Factor analysis: A statistical technique that takes a mass of data and finds hidden patterns shows how those patterns overlap and what characteristics are seen in multiple patterns
False discovery rate: A statistical method of conceptualizing the rate of type I errors in null hypothesis testing when conducting multiple comparisons
Glasgow Coma Scale: A calculated scale that determines a patient's level of consciousness based on the best eye-opening response, the best verbal response, and the best motor response
International Classification of Diseases, 10th Revision, with Canadian Enhancements: A coding system, developed by the World Health Organization that is used to classify diseases and related health problems (morbidity); includes enhancements developed by the Canadian Institute for Health Information for use in Canadian hospitals and other Canadian medical facilities
Heatmaps: Depict relationships between two variables, one plotted on each axis; by observing how cell colors change across each axis, one can observe if there are any patterns in value for one or both variables
National Ambulatory Care Reporting System: Contains data for all hospital-based and community-based ambulatory care: day surgery, outpatient and community-based clinics, emergency departments
Traumatic brain injury: A disruption in the brain's normal function by a bump, blow, or jolt to the head or penetrating head injury
Surveillance (in injury): Refers to the ongoing collection of data describing the occurrence of, and factors associated with, injury
