# Supplementary material for: Decoding health status transitions of over 200 000 patients with traumatic brain injury from preceding injury to the injury event
Source: Sci Rep. 2022 Apr 4;12:5584. doi: 10.1038/s41598-022-08782-0 (PMC8980052; doi:10.1038/s41598-022-08782-0)

**Supplementary Material**

[**Supplementary Table 1: Distribution of characteristics in the TBI cohort.** Note: Frequencies less than 6 are hidden to protect confidentiality. 2](#_Toc92801678)

[**Supplementary Table 2: Scree table of the first 50 factors.** Provides eigenvalues, their differences, and the proportion/cumulative proportion of total variance explained by each factor. Examination of the table was used to decide the optimal number of factors. 3](#_Toc92801679)

[**Supplementary Table 3: Distribution of ICD-10-CA codes in traumatic brain injury patients with an unspecified injury severity.** 6](#_Toc92801680)

[**Supplementary Table 4: Frequencies, ORs, and factor loadings of codes that met the factor analysis cut-off.** 7](#_Toc92801681)

[**Supplementary Table 5: Frequencies, ORs, and factor loadings of codes that did not meet the factor analysis cut-off.** 13](#_Toc92801682)

[**Supplementary Figure 1: Scree plot of the first 50 factors.** The “elbow” of the plot was a method used to determine the optimal number of factors. 15](#_Toc92801683)

[**Supplementary Figure 2: Training and validation set heatmaps and hierarchical clustering between 43 factors preceding TBI and 34 Factors at the TBI event.** The training set heatmap is shown first and the validation heatmap is shown second. 16](#_Toc92801684)

[**Supplementary Figure 3: Training and validation set heatmaps and hierarchical clustering between 43 factors preceding TBI and mechanism, context of injury and TBI severity.** The training set heatmap is shown first and the validation heatmap is shown second. 17](#_Toc92801685)

[**Supplementary Figure 4: Schematic presentation of the study design.** Health status pattern recognition (cluster analysis) before the index date and associated injury events’ patterns. 18](#_Toc92801686)

# **Supplementary Table 1: Distribution of characteristics in the TBI cohort.** Note: Frequencies less than 6 are hidden to protect confidentiality.

| VARIABLES | TBI PTS, by injury severity (58,516, %) | | | |
| --- | --- | --- | --- | --- |
|  | Unspecified  (25,036, 42.78) | Mild  (20,461, 34.97) | Moderate  (2,166, 3.70) | Severe  (10,853, 18.55) |
| Socio-demographic characteristics | | | | |
| Age at first TBI, years old  Mean (SD)  Median (Q1-Q3) | 30.57 (21.50)  23 (15-45) | 29.31 (21.17)  21 (14-43) | 45.39 (23.94)  43 (24-65) | 60.53 (25.70)  68 (43-82) |
| Income quantile  Q1 (lowest) | 4,664 (18.63) | 3,874 (18.93) | 526 (24.28) | 2,401 (22.12) |
| Q2 | 4,807 (19.20) | 3,964 (19.37) | 453 (20.91) | 2,316 (21.34) |
| Q3 | 4,971 (19.86) | 3,976 (19.43) | 382 (17.64) | 2,165 (19.95) |
| Q4 | 5,429 (21.68) | 4,321 (21.12) | 405 (18.70) | 2,027 (18.68) |
| Q5 (highest) | 5,165 (20.63) | 4,326 (21.14) | 400 (18.47) | 1,944 (17.91) |
| Rurality (yes) | 4,213 (16.83) | 3,193 (15.61) | 270 (12.47) | 1,408 (12.97) |
| Injury-event factors | | | | |
| Factor 1: Multitrauma | 452 (1.81) | 416 (2.03) | 207 (9.56) | 1,460 (13.45) |
| Factor 2: Heart & Metabolic Disorders | 1,102 (4.40) | 739 (3.61) | 255 (11.77) | 3,999 (36.85) |
| Factor 3: Alzheimer’s & Dementia | 30 (0.12) | 20 (0.10) | 9 (0.42) | 208 (1.92) |
| Factor 4: Endocrine, Metabolic & Elderly Emerg | 977 (3.90) | 671 (3.28) | 289 (13.34) | 4,401 (40.55) |
| Factor 5: Complications & Resp Emerg | 67 (0.27) | 44 (0.22) | 24 (1.11) | 503 (4.63) |
| Factor 6:  Elderly Disorders & Neoplasms | 2,871 (11.47) | 2,017 (9.86) | 441 (20.36) | 5,464 (50.35) |
| Factor 7: Stroke & Brain Emerg Sequelae | 107 (0.43) | 64 (0.31) | 38 (1.75) | 720 (6.63) |
| Factor 8: Renal Dysfunction | 108 (0.43) | 76 (0.37) | 32 (1.48) | 629 (5.80) |
| Factor 9: Resp Emerg, Septicemia | 108 (0.43) | 60 (0.29) | 40 (1.85) | 636 (5.86) |
| Factor 10: Coagulopathies | 100 (0.40) | 68 (0.33) | 25 (1.15) | 633 (5.83) |
| Factor 11: Liver disorders | 34 (0.14) | 27 (0.13) | 7 (0.32) | 130 (1.20) |
| Factor 12: Resp Infections & ABX Resistance | 132 (0.53) | 101 (0.49) | 50 (2.31) | 864 (7.96) |
| Factor 13: Airway Obstruction | 239 (0.95) | 137 (0.67) | 71 (3.28) | 1,509 (13.90) |
| Factor 14: Parkinson’s & Dementia | 34 (0.14) | 19 (0.09) | 12 (0.55) | 156 (1.44) |
| Factor 15: Abuse & Sexual Assault | 70 (0.28) | 68 (0.33) | 16 (0.74) | 42 (0.39) |
| Factor 16: Falls & Syncope | 5,577 (22.28) | 4,161 (20.34) | 547 (25.25) | 5,231 (48.20) |
| Factor 17: Diabetic emergencies | 851 (3.40) | 605 (2.96) | 130 (6.00) | 1,893 (17.44) |
| Factor 18: Car Collision | 2,112 (8.44) | 1,466 (7.16) | 171 (7.89) | 974 (8.97) |
| Factor 19: Brain & Other Hemorrhages | 307 (1.23) | 225 (1.10) | 124 (5.72) | 2,231 (20.56) |
| Factor 20: Seizures & Drug Adversities | 256 (1.02) | 170 (0.83) | 44 (2.03) | 814 (7.50) |
| Factor 21: Assault | 31 (0.12) | 44 (0.22) | 12 (0.55) | 91 (0.84) |
| Factor 22: Motorcycle Collision | 632 (2.52) | 589 (2.88) | 130 (6.00) | 782 (7.21) |
| Factor 23: Alcohol & Drugs Misuse | 442 (1.77) | 431 (2.11) | 144 (6.65) | 943 (8.69) |
| Factor 24: Multiple systems collapse | 68 (0.27) | 27 (0.13) | 38 (1.75) | 496 (4.57) |
| Factor 25: Intracranial Pathology, Convalescence | 193 (0.77) | 167 (0.82) | 90 (4.16) | 1,475 (13.59) |
| Factor 26: Assault & Alcohol Disorders | 1,842 (7.36) | 2,159 (10.55) | 716 (33.06) | 1,434 (13.21) |
| Factor 27: Aplastic Anemias & Hemorrhages | 82 (0.33) | 76 (0.37) | 37 (1.71) | 464 (4.28) |
| Factor 28: Risky Behaviors & Social Disparities | 584 (2.33) | 549 (2.68) | 176 (8.13) | 1,108 (10.21) |
| Factor 29: Superficial Injuries | 857 (3.42) | 596 (2.91) | 56 (2.59) | 304 (2.80) |
| Factor 30: Pedal cycle injuries | 874 (3.49) | 911 (4.45) | 103 (4.76) | 404 (3.72) |
| Factor 31: Heavy Transport Injuries | 1,239 (4.95) | 851 (4.16) | 99 (4.57) | 472 (4.35) |
| Factor 32: Pedestrian Injuries, Car Collision | 245 (0.98) | 210 (1.03) | 68 (3.14) | 459 (4.23) |
| Factor 33: Falls from Elevation | 768 (3.07) | 653 (3.19) | 88 (4.06) | 638 (5.88) |
| Factor 34: Headache, Blurred Vision & Object Strikes | 6,228 (24.88) | 4,246 (20.75) | 232 (10.71) | 898 (8.27) |

#

# **Supplementary Table 2: Scree table of the first 50 factors.** Provides eigenvalues, their differences, and the proportion/cumulative proportion of total variance explained by each factor. Examination of the table was used to decide the optimal number of factors.

| Factor | Eigenvalue | Difference | Proportion of Variance | Cumulative Proportion of Variance |
| --- | --- | --- | --- | --- |
| 1 | 4.743 | 2.157 | 0.021 | 0.021 |
| 2 | 2.586 | 0.668 | 0.011 | 0.032 |
| 3 | 1.918 | 0.101 | 0.009 | 0.041 |
| 4 | 1.816 | 0.122 | 0.008 | 0.049 |
| 5 | 1.694 | 0.038 | 0.008 | 0.056 |
| 6 | 1.656 | 0.047 | 0.007 | 0.064 |
| 7 | 1.608 | 0.021 | 0.007 | 0.071 |
| 8 | 1.587 | 0.097 | 0.007 | 0.078 |
| 9 | 1.490 | 0.050 | 0.007 | 0.085 |
| 10 | 1.440 | 0.036 | 0.006 | 0.091 |
| 11 | 1.404 | 0.026 | 0.006 | 0.097 |
| 12 | 1.378 | 0.016 | 0.006 | 0.103 |
| 13 | 1.362 | 0.015 | 0.006 | 0.109 |
| 14 | 1.347 | 0.019 | 0.006 | 0.115 |
| 15 | 1.328 | 0.008 | 0.006 | 0.121 |
| 16 | 1.320 | 0.016 | 0.006 | 0.127 |
| 17 | 1.304 | 0.012 | 0.006 | 0.133 |
| 18 | 1.292 | 0.022 | 0.006 | 0.138 |
| 19 | 1.270 | 0.013 | 0.006 | 0.144 |
| 20 | 1.257 | 0.026 | 0.006 | 0.150 |
| 21 | 1.231 | 0.014 | 0.005 | 0.155 |
| 22 | 1.216 | 0.002 | 0.005 | 0.160 |
| 23 | 1.214 | 0.009 | 0.005 | 0.166 |
| 24 | 1.205 | 0.002 | 0.005 | 0.171 |
| 25 | 1.203 | 0.015 | 0.005 | 0.176 |
| 26 | 1.188 | 0.018 | 0.005 | 0.182 |
| 27 | 1.170 | 0.013 | 0.005 | 0.187 |
| 28 | 1.157 | 0.002 | 0.005 | 0.192 |
| 29 | 1.155 | 0.014 | 0.005 | 0.197 |
| 30 | 1.141 | 0.004 | 0.005 | 0.202 |
| 31 | 1.138 | 0.006 | 0.005 | 0.207 |
| 32 | 1.132 | 0.000 | 0.005 | 0.212 |
| 33 | 1.132 | 0.009 | 0.005 | 0.217 |
| 34 | 1.123 | 0.003 | 0.005 | 0.222 |
| 35 | 1.120 | 0.015 | 0.005 | 0.227 |
| 36 | 1.105 | 0.001 | 0.005 | 0.232 |
| 37 | 1.104 | 0.004 | 0.005 | 0.237 |
| 38 | 1.101 | 0.004 | 0.005 | 0.242 |
| 39 | 1.097 | 0.003 | 0.005 | 0.247 |
| 40 | 1.094 | 0.004 | 0.005 | 0.252 |
| 41 | 1.091 | 0.003 | 0.005 | 0.256 |
| 42 | 1.088 | 0.003 | 0.005 | 0.261 |
| 43 | 1.085 | 0.008 | 0.005 | 0.266 |
| 44 | 1.077 | 0.001 | 0.005 | 0.271 |
| 45 | 1.075 | 0.002 | 0.005 | 0.275 |
| 46 | 1.073 | 0.002 | 0.005 | 0.280 |
| 47 | 1.071 | 0.001 | 0.005 | 0.285 |
| 48 | 1.069 | 0.007 | 0.005 | 0.290 |
| 49 | 1.062 | 0.001 | 0.005 | 0.294 |
| 50 | 1.061 | 0.001 | 0.005 | 0.299 |

# **Supplementary Table 3: Distribution of ICD-10-CA codes in traumatic brain injury patients with an unspecified injury severity.**

| Code | Description | Frequency | Percentage (%) |
| --- | --- | --- | --- |
| S060 | Concussion | 22,783 | 91 |
| S066 | Traumatic subarachnoid hemorrhage | 729 | 2.91 |
| S069 | Unspecified intracranial injury | 655 | 2.62 |
| S02900 | Fracture of skull and facial bones, part unspecified, closed | 524 | 2.09 |
| S0625 | Diffuse brain injury without open intracranial wound | 295 | 1.18 |
| S02901 | Fracture of skull and facial bones, part unspecified, open | 23 | 0.09 |
| S061 | Traumatic cerebral edema | 20-25^†^ | 0.07-0.11^†^ |
| * | * | * | * |
| All |  | **25,036** | **100** |

^†^ Values have been given as a range to prevent back-calculation of small cell sizes
*Values have been suppressed due to small cell size

# **Supplementary Table 4: Frequencies, ORs, and factor loadings of codes that met the factor analysis cut-off.**

| **Factor** | **Description** | | **Frequency** | | **OR [95% CI]** | **P-value** | **Factor Loading** |
| --- | --- | --- | --- | --- | --- | --- | --- |
|  |  |  | **TBI** | **Reference** |  |  |  |
| **1** | **Trauma: Chest, abdominal, upper and lower extremity injuries** | | **2,535** | **749** | **3.49 [3.21, 3.79]** | **<0.0001** | **-** |
|  | S36 | Injury of intra-abdominal organs | 518 | 18 | 28.78 [17.99, 46.04] |  | 0.66 |
|  | S27 | Injury of other and unspecified intrathoracic organs | 1,088 | 14 | 77.71 [45.87, 131.66] |  | 0.66 |
|  | S37 | Injury of urinary and pelvic organs | 189 | 18 | 10.50 [6.47, 17.03] |  | 0.48 |
|  | T06 | Other injuries involving multiple body regions, not elsewhere classified | 353 | 12 | 29.42 [16.55, 52.30] |  | 0.38 |
|  | T79 | Certain early complications of trauma, not elsewhere classified | 209 | 19 | 11.00 [6.88, 17.59] |  | 0.38 |
|  | S42 | Fracture of shoulder and upper arm | 1,074 | 485 | 2.23 [2.00, 2.49] |  | 0.3 |
|  | S26 | Injury of heart | NR | <6 | 25.50 [6.21, 104.74] |  | 0.3 |
|  | S72 | Fracture of femur | 421 | 192 | 2.22 [1.87, 2.64] |  | 0.23 |
| **2** | **Cardiology and Disorders of Metabolism: Cardiac disease and metabolic insufficiencies** | | **6,095** | **3,787** | **1.97 [1.88, 2.07]** | **<0.0001** | **-** |
|  | E78 | Disorders of lipoprotein metabolism and other lipidemias | 478 | 302 | 1.61 [1.39, 1.86] |  | 0.59 |
|  | I10 | Essential (primary) hypertension | 3,283 | 1,870 | 1.97 [1.85, 2.10] |  | 0.54 |
|  | I48 | Atrial fibrillation and flutter | 1,333 | 547 | 2.59 [2.33, 2.87] |  | 0.54 |
|  | Z95 | Presence of cardiac and vascular implants and grafts | 558 | 278 | 2.05 [1.77, 2.38] |  | 0.53 |
|  | I50 | Heart failure | 509 | 403 | 1.28 [1.12, 1.46] |  | 0.44 |
|  | Z92 | Personal history of medical treatment | 646 | 122 | 5.44 [4.47, 6.62] |  | 0.34 |
|  | E03 | Other hypothyroidism | 204 | 131 | 1.57 [1.26, 1.96] |  | 0.3 |
|  | E11 | Type 2 diabetes mellitus | 2,813 | 1,699 | 1.80 [1.69, 1.92] |  | 0.23 |
| **3** | **Neurology/Behavioural Neurology: Alzheimer's disease and dementia** | | **267** | **79** | **3.41 [2.65, 4.39]** | **<0.0001** | **-** |
|  | F00 | Dementia in Alzheimer disease | 221 | 62 | 3.56 [2.69, 4.72] |  | 0.95 |
|  | G30 | Alzheimer's disease | 265 | 78 | 3.43 [2.66, 4.42] |  | 0.95 |
| **4** | **Emergency Medicine: Emergencies involving the nervous system, other emergencies** | | **6,338** | **2,873** | **2.85 [2.70, 3.00]** | **<0.0001** | **-** |
|  | E87 | Other disorders of fluid, electrolyte and acid-base balance | 1,064 | 401 | 2.74 [2.44, 3.08] |  | 0.55 |
|  | E83 | Disorders of mineral metabolism | 113 | 54 | 2.09 [1.51, 2.89] |  | 0.43 |
|  | E22 | Hyperfunction of pituitary gland | 114 | 8 | 14.25 [6.96, 29.18] |  | 0.39 |
|  | F05 | Delirium, not induced by alcohol and other psychoactive substances | 689 | 135 | 5.50 [4.54, 6.67] |  | 0.36 |
|  | Z75 | Problems related to medical facilities and other health care | 2,189 | 402 | 6.27 [5.59, 7.03] |  | 0.32 |
|  | N17 | Acute kidney failure | 438 | 259 | 1.72 [1.47, 2.01] |  | 0.31 |
|  | F06 | Other mental disorders due to known physiological condition | 83 | 23 | 3.61 [2.27, 5.73] |  | 0.29 |
|  | B96 | Other bacterial agents as the cause of diseases classified elsewhere | 823 | 377 | 2.27 [2.00, 2.57] |  | 0.23 |
|  | I95 | Hypotension | 425 | 139 | 3.13 [2.58, 3.81] |  | 0.22 |
|  | R41 | Other symptoms and signs involving cognitive functions and awareness | 980 | 185 | 5.52 [4.70, 6.48] |  | 0.21 |
|  | I10 | Essential (primary) hypertension | 3,283 | 1,870 | 1.97 [1.85, 2.10] |  | 0.2 |
| **5** | **Emergency Medicine: Complications of medical procedures** | | **638** | **204** | **3.14 [2.68, 3.67]** | **<0.0001** | **-** |
|  | J95 | Intraoperative and postprocedural complications and disorders of respiratory system, not elsewhere classified | 310 | 35 | 8.86 [6.24, 12.56] |  | 0.75 |
|  | Y84 | Other medical procedures as the cause of abnormal reaction of the patient, or of later complication, without mention of misadventure at the time of the procedure | 453 | 151 | 3.00 [2.50, 3.61] |  | 0.72 |
|  | J15 | Bacterial pneumonia, not elsewhere classified | 176 | 28 | 6.29 [4.22, 9.37] |  | 0.53 |
| **6** | **Neurology/Oncology: Disorders of frailty, neoplastic diseases, and dementia** | | **10,793** | **2,275** | **6.92 [6.54, 7.31]** | **<0.0001** | **-** |
|  | F03 | Unspecified dementia | 634 | 179 | 3.88 [3.26, 4.62] |  | 0.46 |
|  | Z51 | Encounter for other aftercare | 1,269 | 529 | 2.49 [2.25, 2.77] |  | 0.38 |
|  | R29 | Other symptoms and signs involving the nervous and musculoskeletal systems | 644 | 103 | 6.95 [5.57, 8.65] |  | 0.38 |
|  | Z75 | Problems related to medical facilities and other health care | 2,189 | 402 | 6.27 [5.59, 7.03] |  | 0.29 |
|  | W05 | Fall from non-moving wheelchair, nonmotorized scooter and motorized mobility scooter | 440 | 29 | 16.81 [11.32, 24.97] |  | 0.29 |
|  | R26 | Abnormalities of gait and mobility | 173 | 35 | 4.94 [3.44, 7.11] |  | 0.27 |
|  | W19 | Unspecified fall | 6,773 | 884 | 9.00 [8.34, 9.71] |  | 0.26 |
|  | C79 | Secondary malignant neoplasm of other and unspecified sites | 110 | 57 | 1.95 [1.41, 2.69] |  | 0.25 |
|  | F05 | Delirium, not induced by alcohol and other psychoactive substances | 689 | 135 | 5.50 [4.54, 6.67] |  | 0.25 |
|  | Z74 | Problems related to care provider dependency | 89 | 34 | 2.62 [1.76, 3.89] |  | 0.23 |
|  | S72 | Fracture of femur | 421 | 192 | 2.22 [1.87, 2.64] |  | 0.22 |
|  | R41 | Other symptoms and signs involving cognitive functions and awareness | 980 | 185 | 5.52 [4.70, 6.48] |  | 0.22 |
|  | W06 | Fall from bed | 805 | 58 | 14.58 [11.10, 19.16] |  | 0.22 |
| **7** | **Emergency Medicine/Neurology: Stroke** | | **929** | **322** | **2.96 [2.61, 3.37]** | **<0.0001** | **-** |
|  | G81 | Hemiplegia and hemiparesis | 251 | 33 | 7.81 [5.41, 11.29] |  | 0.67 |
|  | R47 | Speech disturbances, not elsewhere classified | 220 | 37 | 6.23 [4.36, 8.90] |  | 0.59 |
|  | I63 | Cerebral infarction | 244 | 120 | 2.04 [1.64, 2.54] |  | 0.5 |
|  | I69 | Sequelae of cerebrovascular disease | 167 | 28 | 6.15 [4.09, 9.23] |  | 0.45 |
|  | R13 | Aphagia and dysphagia | 291 | 166 | 1.76 [1.45, 2.13] |  | 0.31 |
| **8** | **Nephrology: Renal issues** | | **845** | **439** | **2.00 [1.77, 2.25]** | **<0.0001** | **-** |
|  | Z99 | Dependence on enabling machines and devices, not elsewhere classified | 82 | 17 | 4.82 [2.86, 8.13] |  | 0.68 |
|  | N18 | Chronic kidney disease (CKD) | 332 | 177 | 1.91 [1.59, 2.30] |  | 0.66 |
|  | N08 | Glomerular disorders in diseases classified elsewhere | 238 | 77 | 3.15 [2.43, 4.08] |  | 0.64 |
|  | N17 | Acute kidney failure | 438 | 259 | 1.72 [1.47, 2.01] |  | 0.32 |
| **9** | **Nephrology/Respirology/Emergency Medicine: Pulmonary, renal, and systemic emergencies** | | **844** | **378** | **2.32 [2.05, 2.63]** | **<0.0001** | **-** |
|  | J17 | Pneumonia in diseases classified elsewhere | 67 | 17 | 3.94 [2.31, 6.71] |  | 0.79 |
|  | A41 | Other sepsis | 277 | 105 | 2.65 [2.12, 3.33] |  | 0.78 |
|  | N17 | Acute kidney failure | 438 | 259 | 1.72 [1.47, 2.01] |  | 0.26 |
|  | J96 | Respiratory failure, not elsewhere classified | 250 | 61 | 4.20 [3.16, 5.58] |  | 0.23 |
| **10** | **Hematology: Coagulation disorders** | | **826** | **183** | **4.65 [3.95, 5.48]** | **<0.0001** | **-** |
|  | Y44 | Agents primarily affecting blood constituents | 150 | 39 | 3.85 [2.70, 5.47] |  | 0.82 |
|  | D68 | Other coagulation defects | 174 | 44 | 3.95 [2.84, 5.50] |  | 0.8 |
|  | Z92 | Personal history of medical treatment | 646 | 122 | 5.44 [4.47, 6.62] |  | 0.27 |
| **11** | **Gastroenterology: Liver disorders** | | **198** | **71** | **2.79 [2.13, 3.66]** | **<0.0001** | **-** |
|  | K70 | Alcoholic liver disease | 110 | 18 | 6.11 [3.71, 10.06] |  | 0.7 |
|  | R18 | Ascites | 43 | 30 | 1.43 [0.90, 2.28] |  | 0.68 |
|  | K72 | Hepatic failure, not elsewhere classified | 42 | 14 | 3.00 [1.64, 5.49] |  | 0.58 |
|  | B18 | Chronic viral hepatitis | 53 | 20 | 2.65 [1.58, 4.43] |  | 0.27 |
| **12** | **Infectious Diseases: Acute infection and treatment resistance** | | **1,147** | **570** | **2.08 [1.88, 2.31]** | **<0.0001** | **-** |
|  | B95 | Streptococcus, Staphylococcus, and Enterococcus as the cause of diseases classified elsewhere | 238 | 186 | 1.28 [1.06, 1.55] |  | 0.72 |
|  | U82 | Resistance to betalactam antibiotics | 94 | 25 | 3.76 [2.42, 5.84] |  | 0.71 |
|  | B96 | Other bacterial agents as the cause of diseases classified elsewhere | 823 | 377 | 2.27 [2.00, 2.57] |  | 0.34 |
|  | A49 | Bacterial infection of unspecified site | 80 | 37 | 2.19 [1.48, 3.25] |  | 0.33 |
|  | L89 | Pressure ulcer | 143 | 20 | 7.15 [4.48, 11.42] |  | 0.21 |
| **13** | **Emergency Medicine: Foreign body in airway and other** | | **1,956** | **771** | **2.70 [2.48, 2.95]** | **<0.0001** | **-** |
|  | J69 | Pneumonitis due to solids and liquids | 359 | 42 | 8.93 [6.44, 12.37] |  | 0.61 |
|  | W80 | Inhalation and ingestion of other objects causing obstruction of respiratory tract | NR | <6 | 16.00 [3.83, 66.76] |  | 0.52 |
|  | J96 | Respiratory failure, not elsewhere classified | 250 | 61 | 4.20 [3.16, 5.58] |  | 0.39 |
|  | R13 | Aphagia and dysphagia | 291 | 166 | 1.76 [1.45, 2.13] |  | 0.27 |
|  | Z51 | Encounter for other aftercare | 1,269 | 529 | 2.49 [2.25, 2.77] |  | 0.23 |
|  | L89 | Pressure ulcer | 143 | 20 | 7.15 [4.48, 11.42] |  | 0.22 |
| **14** | **Neurology: Parkinson's disease and dementia** | | **221** | **50** | **4.42 [3.25, 6.01]** | **<0.0001** | **-** |
|  | F02 | Dementia in other diseases classified elsewhere | 54 | 8 | 6.75 [3.21, 14.18] |  | 0.79 |
|  | G20 | Parkinson disease | 179 | 38 | 4.71 [3.32, 6.69] |  | 0.75 |
|  | G31 | Other degenerative diseases of nervous system, not elsewhere classified | 41 | 11 | 3.73 [1.92, 7.25] |  | 0.41 |
| **15** | **Trauma: Adult and child abuse, and sexual assault** | | **196** | **15** | **13.07 [7.73, 22.09]** | **<0.0001** | **-** |
|  | T74 | Adult and child abuse, neglect and other maltreatment, confirmed | NR | <6 | 10.00 [3.99, 25.08] |  | 0.8 |
|  | Y07 | Perpetrator of assault, maltreatment and neglect | 160 | 8 | 20.00 [9.83, 40.68] |  | 0.69 |
|  | Y05 | Sexual assault by bodily force | 17 | 8 | 2.12 [0.92, 4.92] |  | 0.44 |
|  | Y06 | Neglect and abandonment | NR | <6 | NA [NA, NA] |  | 0.22 |
| **16** | **Emergency Medicine: Emergencies involving the nervous system** | | **15,516** | **3,106** | **6.79 [6.48, 7.10]** | **<0.0001** | **-** |
|  | R55 | Syncope and collapse | 1,368 | 737 | 1.88 [1.72, 2.06] |  | 0.59 |
|  | W18 | Other slipping, tripping and stumbling and falls | 4,547 | 486 | 10.29 [9.33, 11.36] |  | 0.58 |
|  | W19 | Unspecified fall | 6,773 | 884 | 9.00 [8.34, 9.71] |  | 0.38 |
|  | W01 | Fall on same level from slipping, tripping and stumbling | 5,732 | 1,016 | 6.17 [5.75, 6.62] |  | 0.23 |
|  | I95 | Hypotension | 425 | 139 | 3.13 [2.58, 3.81] |  | 0.22 |
| **17** | **Endocrinology: Diabetes and diabetic consequences** | | **3,479** | **2,426** | **1.54 [1.45, 1.63]** | **<0.0001** | **-** |
|  | E11 | Type 2 diabetes mellitus | 2,813 | 1,699 | 1.80 [1.69, 1.92] |  | 0.61 |
|  | E14 | Unspecified diabetes mellitus | 1,090 | 858 | 1.29 [1.17, 1.41] |  | 0.57 |
|  | R73 | Elevated blood glucose level | 209 | 139 | 1.51 [1.22, 1.88] |  | 0.52 |
|  | G63 | Polyneuropathy in diseases classified elsewhere | 40 | 13 | 3.08 [1.65, 5.75] |  | 0.32 |
|  | N08 | Glomerular disorders in diseases classified elsewhere | 238 | 77 | 3.15 [2.43, 4.08] |  | 0.26 |
| **18** | **Trauma: Car accident injuries** | | **4,723** | **711** | **7.14 [6.58, 7.75]** | **<0.0001** | **-** |
|  | V43 | Car occupant injured in collision with car, pick-up truck or van | 2,298 | 255 | 9.48 [8.30, 10.82] |  | 0.46 |
|  | V49 | Car occupant injured in other and unspecified transport accidents | 627 | 36 | 17.89 [12.72, 25.14] |  | 0.46 |
|  | V48 | Car occupant injured in noncollision transport accident | 783 | 48 | 16.31 [12.19, 21.83] |  | 0.42 |
|  | V89 | Motor- or nonmotor-vehicle accident, type of vehicle unspecified | 443 | 34 | 13.03 [9.19, 18.47] |  | 0.4 |
|  | V47 | Car occupant injured in collision with fixed or stationary object | 562 | 28 | 20.07 [13.73, 29.34] |  | 0.33 |
|  | T14 | Injury of unspecified body region | 289 | 123 | 2.35 [1.90, 2.90] |  | 0.29 |
|  | Z04 | Encounter for examination and observation for other reasons | 419 | 232 | 1.81 [1.54, 2.12] |  | 0.24 |
|  | V58 | Occupant of pick-up truck or van injured in noncollision transport accident | 161 | 10 | 16.10 [8.50, 30.50] |  | 0.21 |
| **19** | **Emergency Medicine: Brain hemorrhage** | | **2,887** | **744** | **4.24 [3.90, 4.62]** | **<0.0001** | **-** |
|  | I61 | Nontraumatic intracerebral hemorrhage | 216 | 17 | 12.71 [7.76, 20.82] |  | 0.43 |
|  | I60 | Nontraumatic subarachnoid hemorrhage | 188 | 15 | 12.53 [7.41, 21.21] |  | 0.39 |
|  | G91 | Hydrocephalus | 108 | 11 | 9.82 [5.28, 18.26] |  | 0.35 |
|  | I62 | Other and unspecified nontraumatic intracranial hemorrhage | 772 | 12 | 64.33 [36.38, 113.78] |  | 0.32 |
|  | C79 | Secondary malignant neoplasm of other and unspecified sites | 110 | 57 | 1.95 [1.41, 2.69] |  | 0.26 |
|  | G06 | Intracranial and intraspinal abscess and granuloma | NR | <6 | 4.40 [1.67, 11.62] |  | 0.26 |
|  | Z51 | Encounter for other aftercare | 1,269 | 529 | 2.49 [2.25, 2.77] |  | 0.25 |
|  | I67 | Other cerebrovascular diseases | 79 | 17 | 4.65 [2.75, 7.85] |  | 0.23 |
|  | Z54 | Convalescence | 603 | 117 | 5.23 [4.28, 6.38] |  | 0.22 |
| **20** | **Neurology/Pharmacology Emergencies: Epilepsy, seizures, and prescription drugs' poisoning** | | **1,284** | **288** | **4.56 [4.00, 5.19]** | **<0.0001** | **-** |
|  | Y46 | Antiepileptics and antiparkinsonism drugs | NR | <6 | 19.33 [6.06, 61.70] |  | 0.58 |
|  | G40 | Epilepsy and recurrent seizures | 369 | 76 | 4.91 [3.83, 6.29] |  | 0.49 |
|  | T42 | Poisoning by, adverse effect of and underdosing of antiepileptic, sedative- hypnotic and antiparkinsonism drugs | 59 | 25 | 2.36 [1.48, 3.77] |  | 0.45 |
|  | R56 | Convulsions, not elsewhere classified | 864 | 185 | 4.75 [4.05, 5.58] |  | 0.42 |
|  | G41 | Status epilepticus | 55 | 8 | 6.88 [3.27, 14.43] |  | 0.39 |
|  | R27 | Other lack of coordination | 51 | 13 | 3.92 [2.13, 7.21] |  | 0.21 |
| **21** | **Trauma: Assault by sharp object to upper body regions** | | **178** | **33** | **5.39 [3.72, 7.82]** | **<0.0001** | **-** |
|  | X99 | Assault by sharp object | 92 | 16 | 5.75 [3.38, 9.78] |  | 0.74 |
|  | S21 | Open wound of thorax | 30 | 9 | 3.33 [1.58, 7.02] |  | 0.68 |
|  | S11 | Open wound of neck | 39 | 11 | 3.55 [1.82, 6.92] |  | 0.4 |
|  | S15 | Injury of blood vessels at neck level | NR | <6 | NA [NA, NA] |  | 0.22 |
| **22** | **Trauma: Motorcycle injuries** | | **2,133** | **674** | **3.27 [3.00, 3.58]** | **<0.0001** | **-** |
|  | V28 | Motorcycle rider injured in noncollision transport accident | 252 | 58 | 4.34 [3.27, 5.78] |  | 0.63 |
|  | V29 | Motorcycle rider injured in other and unspecified transport accidents | 90 | 14 | 6.43 [3.66, 11.29] |  | 0.59 |
|  | V27 | Motorcycle rider injured in collision with fixed or stationary object | 51 | 6 | 8.50 [3.65, 19.81] |  | 0.47 |
|  | V23 | Motorcycle rider injured in collision with car, pick-up truck or van | 142 | 16 | 8.88 [5.29, 14.88] |  | 0.31 |
|  | S42 | Fracture of shoulder and upper arm | 1,074 | 485 | 2.23 [2.00, 2.49] |  | 0.24 |
|  | V86 | Occupant of special all-terrain or other off-road motor vehicle, injured in transport accident | 742 | 121 | 6.45 [5.29, 7.85] |  | 0.21 |
|  | V22 | Motorcycle rider injured in collision with two- or three-wheeled motor vehicle | NR | <6 | 20.00 [2.68, 149.03] |  | 0.2 |
| **23** | **Neurology/Psychiatry: Alcohol- and drug-related disorders** | | **1,960** | **435** | **4.66 [4.19, 5.18]** | **<0.0001** | **-** |
|  | Y90 | Evidence of alcohol involvement determined by blood alcohol level | NR | <6 | 26.50 [9.77, 71.91] |  | 0.78 |
|  | R78 | Findings of drugs and other substances, not normally found in blood | 115 | 33 | 3.48 [2.37, 5.13] |  | 0.74 |
|  | F10 | Alcohol related disorders | 1,819 | 402 | 4.67 [4.18, 5.21] |  | 0.23 |
| **24** | **Emergency Medicine: Brain and cardiopulmonary emergencies** | | **629** | **76** | **8.28 [6.52, 10.50]** | **<0.0001** | **-** |
|  | G93 | Other disorders of brain | 413 | 20 | 20.65 [13.18, 32.35] |  | 0.53 |
|  | Z52 | Donors of organs and tissues | 24 | 7 | 3.43 [1.48, 7.96] |  | 0.51 |
|  | D65 | Disseminated intravascular coagulation [defibrination syndrome] | NR | <6 | 7.00 [1.59, 30.80] |  | 0.5 |
|  | E23 | Hypofunction and other disorders of the pituitary gland | NR | <6 | 7.80 [3.07, 19.79] |  | 0.38 |
|  | I46 | Cardiac arrest | 213 | 45 | 4.73 [3.43, 6.53] |  | 0.26 |
| **25** | **Trauma: Fractures of lower limb and multiple regions, and intracranial hemorrhage** | | **1,925** | **363** | **5.75 [5.11, 6.46]** | **<0.0001** | **-** |
|  | Z54 | Convalescence | 603 | 117 | 5.23 [4.28, 6.38] |  | 0.43 |
|  | Z50 | Care involving use of rehabilitation procedures | 410 | 66 | 6.38 [4.90, 8.30] |  | 0.33 |
|  | I62 | Other and unspecified nontraumatic intracranial hemorrhage | 772 | 12 | 64.33 [36.38, 113.78] |  | 0.3 |
|  | S72 | Fracture of femur | 421 | 192 | 2.22 [1.87, 2.64] |  | 0.25 |
| **26** | **Trauma: Assault, injuries to orbits, and alcohol-related disorders** | | **6,151** | **762** | **9.30 [8.58, 10.09]** | **<0.0001** | **-** |
|  | Y04 | Assault by bodily force | 3,796 | 93 | 47.29 [37.89, 59.01] |  | 0.59 |
|  | Y09 | Assault by unspecified means | 375 | 6 | 93.25 [34.81, 249.77] |  | 0.47 |
|  | Y00 | Assault by blunt object | NR | <6 | 145.67 [46.80, 453.41] |  | 0.37 |
|  | H05 | Disorders of orbit | 80 | 37 | 2.16 [1.46, 3.19] |  | 0.26 |
|  | H11 | Other disorders of conjunctiva | 108 | 78 | 1.38 [1.03, 1.85] |  | 0.25 |
|  | H53 | Visual disturbances | 245 | 138 | 1.78 [1.44, 2.19] |  | 0.23 |
|  | F10 | Alcohol related disorders | 1,819 | 402 | 4.67 [4.18, 5.21] |  | 0.21 |
|  | Y08 | Assault by other specified means | 96 | 10 | 9.60 [5.01, 18.41] |  | 0.21 |
| **27** | **Hematology: Bone marrow anemias, and disorders of blood coagulation** | | **659** | **455** | **1.46 [1.29, 1.65]** | **<0.0001** | **-** |
|  | D46 | Myelodysplastic syndromes | NR | <6 | 9.75 [3.48, 27.28] |  | 0.63 |
|  | D61 | Other aplastic anemias and other bone marrow failure syndromes | 54 | 18 | 3.00 [1.76, 5.11] |  | 0.59 |
|  | D69 | Purpura and other hemorrhagic conditions | 144 | 85 | 1.69 [1.30, 2.22] |  | 0.54 |
|  | D64 | Other anemias | 482 | 360 | 1.35 [1.17, 1.55] |  | 0.2 |
| **28** | **Psychiatry/Community Health: Functional and economic inquiry, HIV infection, and substance abuse** | | **2,417** | **694** | **3.64 [3.34, 3.97]** | **<0.0001** | **-** |
|  | F14 | Cocaine related disorders | 78 | 19 | 4.11 [2.49, 6.78] |  | 0.48 |
|  | F11 | Opioid related disorders | 73 | 31 | 2.35 [1.55, 3.58] |  | 0.47 |
|  | Z59 | Problems related to housing and economic circumstances | 42 | 19 | 2.21 [1.29, 3.80] |  | 0.4 |
|  | F10 | Alcohol related disorders | 1,819 | 402 | 4.67 [4.18, 5.21] |  | 0.32 |
|  | Z91 | Personal risk factors, not elsewhere classified | 142 | 72 | 1.97 [1.49, 2.62] |  | 0.32 |
|  | Z72 | Problems related to lifestyle | 431 | 157 | 2.79 [2.32, 3.36] |  | 0.26 |
|  | B18 | Chronic viral hepatitis | 53 | 20 | 2.65 [1.58, 4.43] |  | 0.25 |
|  | Z21 | Asymptomatic human immunodeficiency virus [HIV] infection status | 27 | 8 | 3.38 [1.53, 7.43] |  | 0.25 |
| **29** | **Trauma: Superficial injuries** | | **1,813** | **894** | **2.06 [1.90, 2.23]** | **<0.0001** | **-** |
|  | S30 | Superficial injury of abdomen, lower back, pelvis and external genitals | 363 | 149 | 2.45 [2.02, 2.96] |  | 0.47 |
|  | S40 | Superficial injury of shoulder and upper arm | 390 | 218 | 1.79 [1.52, 2.12] |  | 0.41 |
|  | S70 | Superficial injury of hip and thigh | 206 | 111 | 1.86 [1.48, 2.35] |  | 0.38 |
|  | S20 | Superficial injury of thorax | 479 | 219 | 2.20 [1.88, 2.59] |  | 0.32 |
|  | S10 | Superficial injury of neck | 350 | 82 | 4.27 [3.36, 5.43] |  | 0.32 |
|  | S39 | Other and unspecified injuries of abdomen, lower back, pelvis and external genitals | 235 | 153 | 1.54 [1.26, 1.89] |  | 0.23 |
| **30** | **Trauma: Pedal cycle injuries** | | **2,292** | **514** | **4.64 [4.21, 5.12]** | **<0.0001** | **-** |
|  | V18 | Pedal cycle rider injured in noncollision transport accident | 1,258 | 344 | 3.74 [3.32, 4.23] |  | 0.58 |
|  | V19 | Pedal cycle rider injured in other and unspecified transport accidents | 199 | 53 | 3.75 [2.77, 5.08] |  | 0.53 |
|  | T00 | Superficial injuries involving multiple body regions | 586 | 99 | 5.97 [4.82, 7.39] |  | 0.36 |
|  | V13 | Pedal cycle rider injured in collision with car, pick-up truck or van | 340 | 28 | 12.14 [8.26, 17.85] |  | 0.35 |
|  | V17 | Pedal cycle rider injured in collision with fixed or stationary object | NR | <6 | 19.20 [7.81, 47.18] |  | 0.22 |
| **31** | **Trauma: Heavy machinery motor-vehicle accidents** | | **2,661** | **298** | **9.53 [8.42, 10.79]** | **<0.0001** | **-** |
|  | V44 | Car occupant injured in collision with heavy transport vehicle or bus | 145 | 7 | 20.71 [9.70, 44.23] |  | 0.47 |
|  | V54 | Occupant of pick-up truck or van injured in collision with heavy transport vehicle or bus | NR | <6 | 26.00 [3.53, 191.60] |  | 0.43 |
|  | V43 | Car occupant injured in collision with car, pick-up truck or van | 2,298 | 255 | 9.48 [8.30, 10.82] |  | 0.26 |
|  | S19 | Other specified and unspecified injuries of neck | 297 | 50 | 6.15 [4.53, 8.34] |  | 0.23 |
| **32** | **Trauma: Pedestrian injuries and fractures, induced by transport machinery** | | **982** | **92** | **10.89 [8.77, 13.51]** | **<0.0001** | **-** |
|  | V03 | Pedestrian injured in collision with car, pick-up truck or van | 812 | 61 | 13.73 [10.54, 17.88] |  | 0.46 |
|  | V09 | Pedestrian injured in other and unspecified transport accidents | 64 | 8 | 8.00 [3.84, 16.68] |  | 0.43 |
|  | S15 | Injury of blood vessels at neck level | NR | <6 | NA [NA, NA] |  | 0.32 |
|  | T08 | Fracture of spine, level unspecified | NR | <6 | 2.20 [0.76, 6.33] |  | 0.27 |
|  | T02 | Fractures involving multiple body regions | NR | <6 | 21.33 [6.70, 67.90] |  | 0.25 |
|  | I72 | Other aneurysm | 42 | 17 | 2.47 [1.41, 4.34] |  | 0.2 |
| **33** | **Trauma: Falls from elevation** | | **2,147** | **296** | **7.59 [6.70, 8.59]** | **<0.0001** | **-** |
|  | W17 | Other fall from one level to another | 1,235 | 228 | 5.58 [4.83, 6.44] |  | 0.58 |
|  | W13 | Fall from, out of or through building or structure | 311 | 18 | 17.28 [10.74, 27.79] |  | 0.51 |
|  | W11 | Fall on and from ladder | 679 | 53 | 13.27 [9.99, 17.64] |  | 0.35 |
|  | W12 | Fall on and from scaffolding | NR | <6 | 49.00 [6.77, 354.86] |  | 0.22 |
| **34** | **Neurology/Trauma: Sensory medical and functional inquiry, and strike against an object** | | **11,604** | **2,079** | **6.80 [6.45, 7.16]** | **<0.0001** | **-** |
|  | R51 | Headache | 1,464 | 573 | 2.61 [2.36, 2.87] |  | 0.51 |
|  | F07 | Personality and behavioral disorders due to known physiological condition | NR | <6 | NA [NA, NA] |  | 0.47 |
|  | G44 | Other headache syndromes | 124 | 50 | 2.48 [1.79, 3.44] |  | 0.32 |
|  | W22 | Striking against or struck by other objects | 8,386 | 1,203 | 8.13 [7.62, 8.68] |  | 0.32 |
|  | Z02 | Encounter for administrative examination | 236 | 65 | 3.67 [2.79, 4.84] |  | 0.21 |
|  | W20 | Struck by thrown, projected or falling object | 1,480 | 206 | 7.43 [6.41, 8.62] |  | 0.2 |

NR = Not Reported due to small cell size; TBI = Traumatic Brain Injury; OR = Odds Ratio; CI = Confidence Interval

# **Supplementary Table 5: Frequencies, ORs, and factor loadings of codes that did not meet the factor analysis cut-off.**

| **Code** | **Description** | **Frequency** | | **OR [95% CI]** |
| --- | --- | --- | --- | --- |
|  |  | **TBI** | **Reference** |  |
| **I. Certain infectious and parasitic diseases** | |  |  |  |
| A04 | Other bacterial intestinal infections | 108 | 56 | 1.93 [1.40, 2.66] |
| **III. Diseases of the blood and blood-forming organs and certain disorders involving the immune mechanism** | |  |  |  |
| D62 | Acute posthaemorrhagic anaemia | 88 | 65 | 1.36 [0.98, 1.88] |
| **V. Mental and behavioural disorders** | |  |  |  |
| F01 | Vascular dementia | 42 | 17 | 2.47 [1.41, 4.34] |
| F44 | Dissociative and conversion disorders | 17 | 7 | 2.43 [1.01, 5.86] |
| **VI. Diseases of the nervous system** | |  |  |  |
| G96 | Other disorders of central nervous system | NR | <6 | 115.00 [16.06, 823.39] |
| **VII. Diseases of the eye and adnexa** | |  |  |  |
| H49 | Paralytic strabismus | NR | <6 | 5.25 [1.80, 15.29] |
| H54 | Blindness and low vision | 35 | 16 | 2.19 [1.21, 3.95] |
| **IX. Diseases of the circulatory system** | |  |  |  |
| I82 | Other venous embolism and thrombosis | 38 | 13 | 2.92 [1.56, 5.49] |
| **X. Diseases of the respiratory system** | |  |  |  |
| J80 | Adult respiratory distress syndrome | 67 | 9 | 7.44 [3.71, 14.93] |
| J90 | Pleural effusion, not elsewhere classified | 163 | 102 | 1.60 [1.25, 2.06] |
| **XIII. Diseases of the musculoskeletal system and connective tissue** | |  |  |  |
| M81 | Osteoporosis without current pathological fracture | 122 | 56 | 2.22 [1.61, 3.06] |
| **XVIII. Symptoms, signs and abnormal clinical and laboratory findings, not elsewhere classified** | |  |  |  |
| R40 | Somnolence, stupor and coma | 119 | 17 | 7.00 [4.21, 11.64] |
| R74 | Abnormal serum enzyme levels | 65 | 33 | 2.00 [1.31, 3.06] |
| **XIX. Injury, poisoning and certain other consequences of external causes** | |  |  |  |
| S16 | Injury of muscle, fascia and tendon at neck level | 85 | 13 | 6.54 [3.65, 11.72] |
| S29 | Other and unspecified injuries of thorax | 126 | 95 | 1.33 [1.02, 1.74] |
| T01 | Open wounds involving multiple body regions | 41 | 11 | 3.73 [1.92, 7.25] |
| T07 | Unspecified multiple injuries | NR | <6 | 30.25 [11.17, 81.91] |
| T09 | Other injuries of spine and trunk, level unspecified | 134 | 66 | 2.03 [1.51, 2.73] |
| **XX. External causes of morbidity and mortality** | |  |  |  |
| V01 | Pedestrian injured in collision with pedal cycle | NR | <6 | 44.00 [6.06, 319.37] |
| V02 | Pedestrian injured in collision with two- or three-wheeled motor vehicle | NR | <6 | NA [NA, NA] |
| V10 | Pedal cycle rider injured in collision with pedestrian or animal | NR | <6 | 14.00 [1.84, 106.47] |
| V11 | Pedal cycle rider injured in collision with other pedal cycle | NR | <6 | 6.60 [2.58, 16.91] |
| V14 | Pedal cycle rider injured in collision with heavy transport vehicle or bus | NR | <6 | 12.00 [1.56, 92.29] |
| V20 | Motorcycle rider injured in collision with pedestrian or animal | NR | <6 | NA [NA, NA] |
| V38 | Occupant of three-wheeled motor vehicle injured in noncollision transport accident | NR | <6 | 13.50 [3.21, 56.77] |
| V40 | Car occupant injured in collision with pedestrian or animal | NR | <6 | 17.00 [5.31, 54.47] |
| V53 | Occupant of pick-up truck or van injured in collision with car, pick-up truck or van | 91 | 15 | 6.07 [3.51, 10.48] |
| V59 | Occupant of pick-up truck or van injured in other and unspecified transport accidents | NR | <6 | NA [NA, NA] |
| V68 | Occupant of heavy transport vehicle injured in noncollision transport accident | NR | <6 | 15.67 [4.88, 50.33] |
| V73 | Bus occupant injured in collision with car, pick-up truck or van | NR | <6 | 7.50 [1.72, 32.80] |
| V78 | Bus occupant injured in noncollision transport accident | 69 | 7 | 9.86 [4.53, 21.45] |
| V80 | Animal-rider or occupant of animal-drawn vehicle injured in transport accident | 472 | 32 | 15.19 [10.56, 21.85] |
| V83 | Occupant of special vehicle mainly used on industrial premises injured in transport accident | NR | <6 | 5.20 [2.00, 13.54] |
| V84 | Occupant of special vehicle mainly used in agriculture injured in transport accident | NR | <6 | 22.00 [2.97, 163.22] |
| V87 | Traffic accident of specified type but victim's mode of transport unknown | NR | <6 | 15.00 [1.98, 113.56] |
| V91 | Other injury due to accident to watercraft | NR | <6 | 13.50 [3.21, 56.77] |
| V92 | Drowning and submersion due to accident on board watercraft, without accident to watercraft | NR | <6 | 15.00 [1.98, 113.56] |
| V93 | Other injury due to accident on board watercraft, without accident to watercraft | NR | <6 | 12.00 [4.82, 29.88] |
| V94 | Other and unspecified water transport accidents | 97 | 9 | 10.78 [5.44, 21.34] |
| W00 | Fall due to ice and snow | 2,355 | 242 | 10.23 [8.93, 11.71] |
| W02 | Fall involving ice-skates, skis, roller-skates or skateboards | 2,515 | 563 | 4.87 [4.42, 5.35] |
| W03 | Other fall on same level due to collision with another person | 273 | 28 | 9.75 [6.61, 14.39] |
| W04 | Fall while being carried or supported by other persons | NR | <6 | 65.75 [24.49, 176.49] |
| W07 | Fall from chair | 568 | 44 | 12.91 [9.50, 17.54] |
| W08 | Fall from other furniture | 326 | 21 | 16.25 [10.35, 25.52] |
| W09 | Fall on and from playground equipment | 437 | 145 | 3.12 [2.57, 3.77] |
| W10 | Fall on and from stairs and steps | 3,602 | 412 | 9.24 [8.32, 10.26] |
| W14 | Fall from tree | 90 | 25 | 3.60 [2.31, 5.61] |
| W15 | Fall from cliff | NR | <6 | NA [NA, NA] |
| W16 | Fall, jump or diving into water | 92 | 14 | 6.57 [3.75, 11.53] |
| W21 | Striking against or struck by sports equipment | 2,226 | 421 | 5.59 [5.02, 6.23] |
| W24 | Contact with lifting and transmission devices, not elsewhere classified | 26 | 7 | 3.71 [1.61, 8.56] |
| W50 | Accidental hit, strike, kick, twist, bite or scratch by another person | 1,052 | 156 | 6.82 [5.76, 8.07] |
| W51 | Accidental striking against or bumped into by another person | 5,219 | 601 | 11.38 [10.33, 12.54] |
| W55 | Contact with other mammals | 197 | 94 | 2.10 [1.64, 2.68] |
| X59 | Exposure to unspecified factor | 2,398 | 1,334 | 1.83 [1.71, 1.95] |
| X83 | Intentional self-harm by other specified means | NR | <6 | 13.00 [1.70, 99.38] |
| Y35 | Legal intervention | NR | <6 | 63.00 [8.74, 454.24] |
| Y85 | Sequelae of transport accidents | NR | <6 | 36.00 [4.94, 262.58] |
| Y86 | Sequelae of other accidents | 53 | 13 | 4.08 [2.22, 7.48] |
| Y91 | Evidence of alcohol involvement determined by level of intoxication | 382 | 12 | 31.83 [17.92, 56.55] |
| **XXI. Factors influencing health status and contact with health services** | |  |  |  |
| Z23 | Need for immunization against single bacterial diseases | 121 | 49 | 2.50 [1.79, 3.49] |

NR = Not Reported due to small cell size; TBI = Traumatic Brain Injury; OR = Odds Ratio; CI = Confidence Interval

# **Supplementary Figure 1: Scree plot of the first 50 factors.** The “elbow” of the plot was a method used to determine the optimal number of factors.


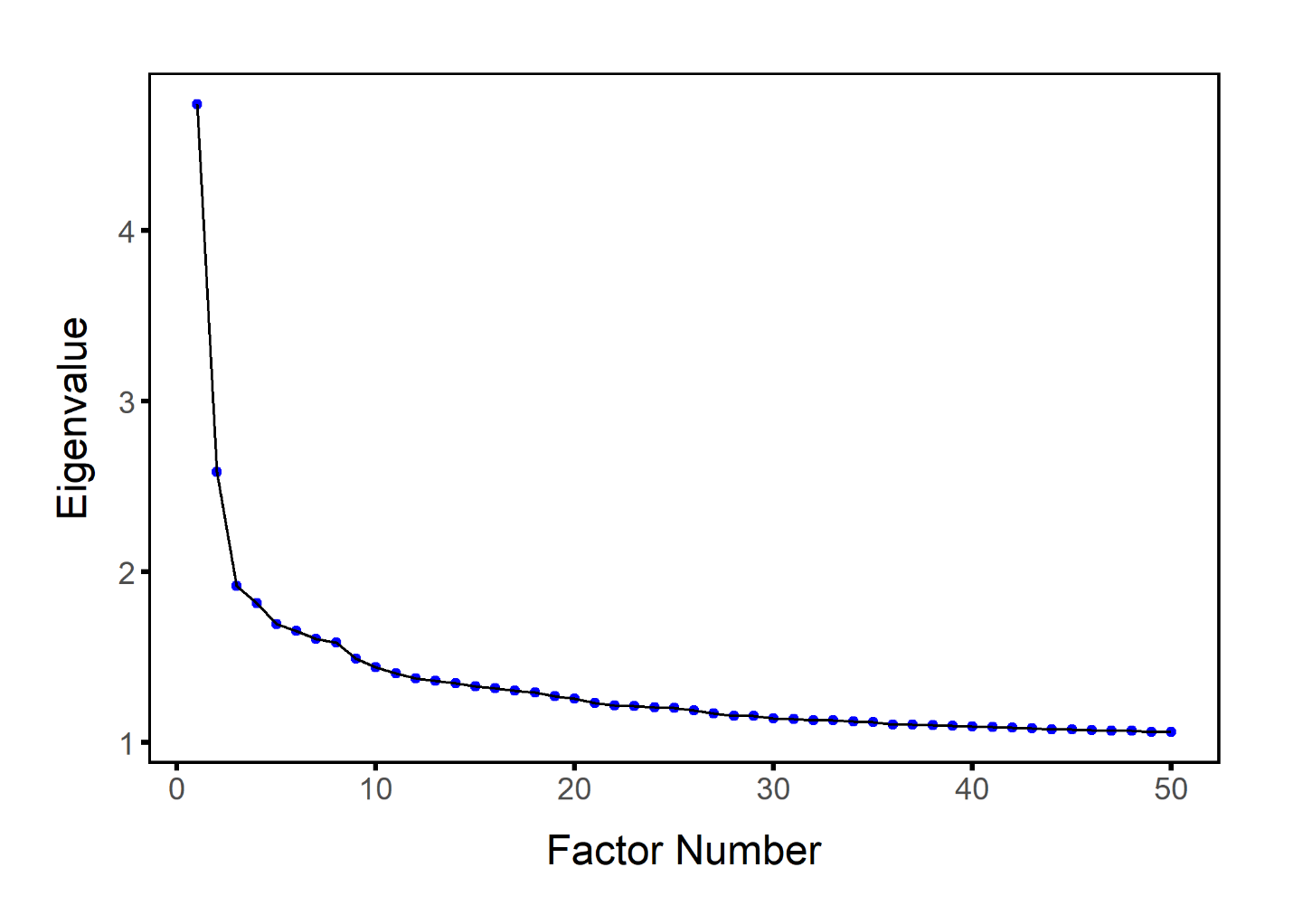


# **Supplementary Figure 2: Training and validation set heatmaps and hierarchical clustering between 43 factors preceding TBI and 34 Factors at the TBI event.** The training set heatmap is shown first and the validation heatmap is shown second.


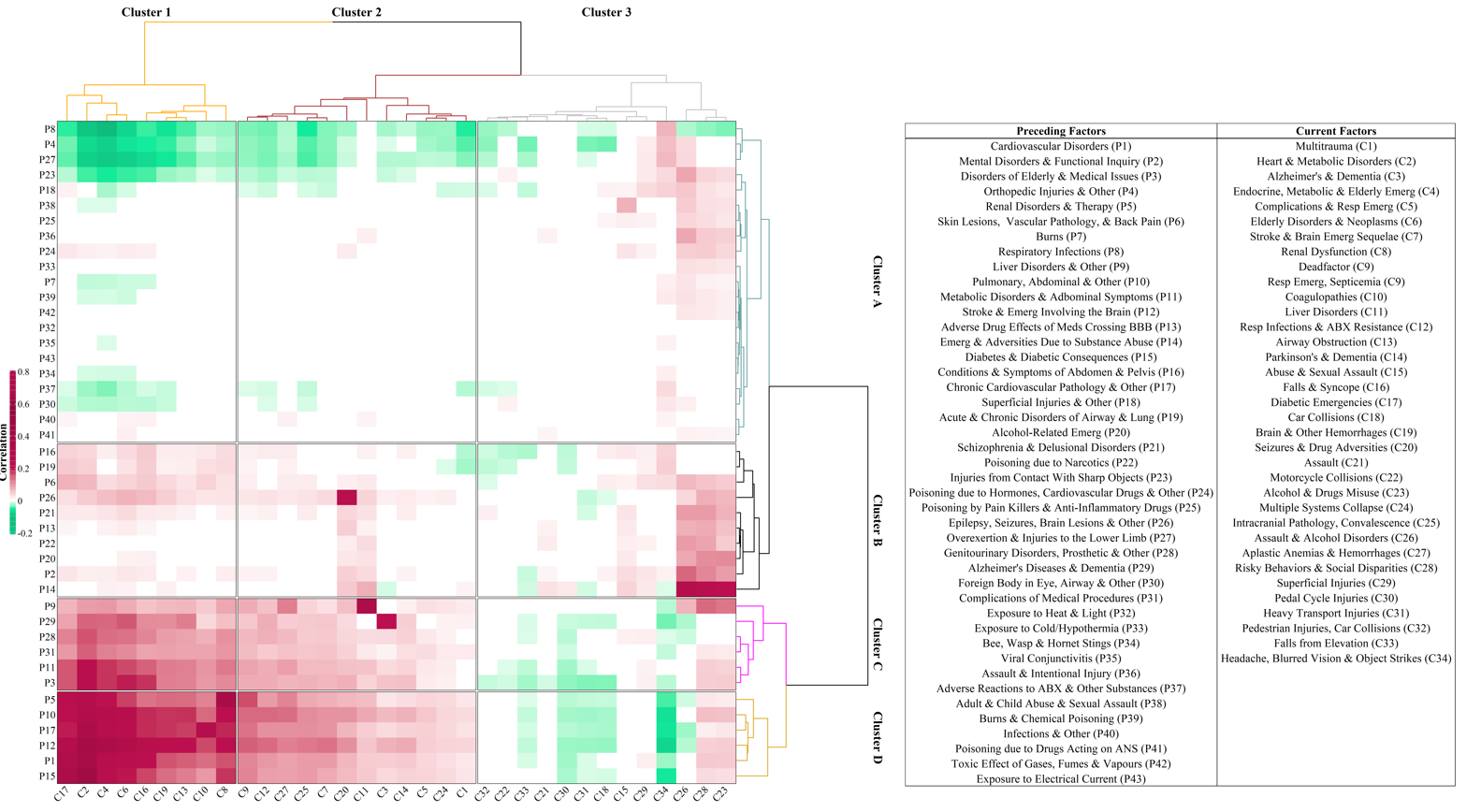


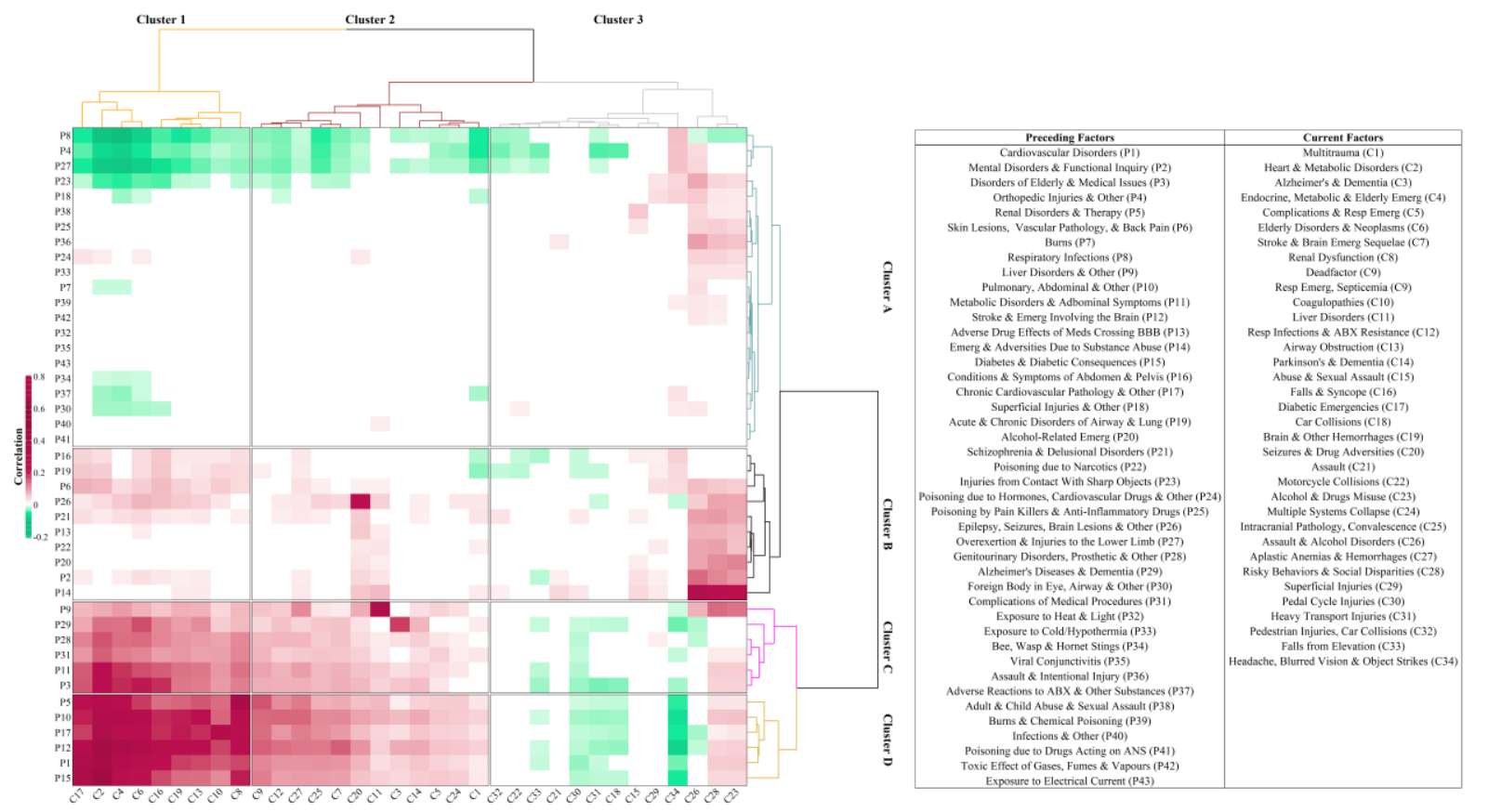


# **Supplementary Figure 3: Training and validation set heatmaps and hierarchical clustering between 43 factors preceding TBI and mechanism, context of injury and TBI severity.** The training set heatmap is shown first and the validation heatmap is shown second.


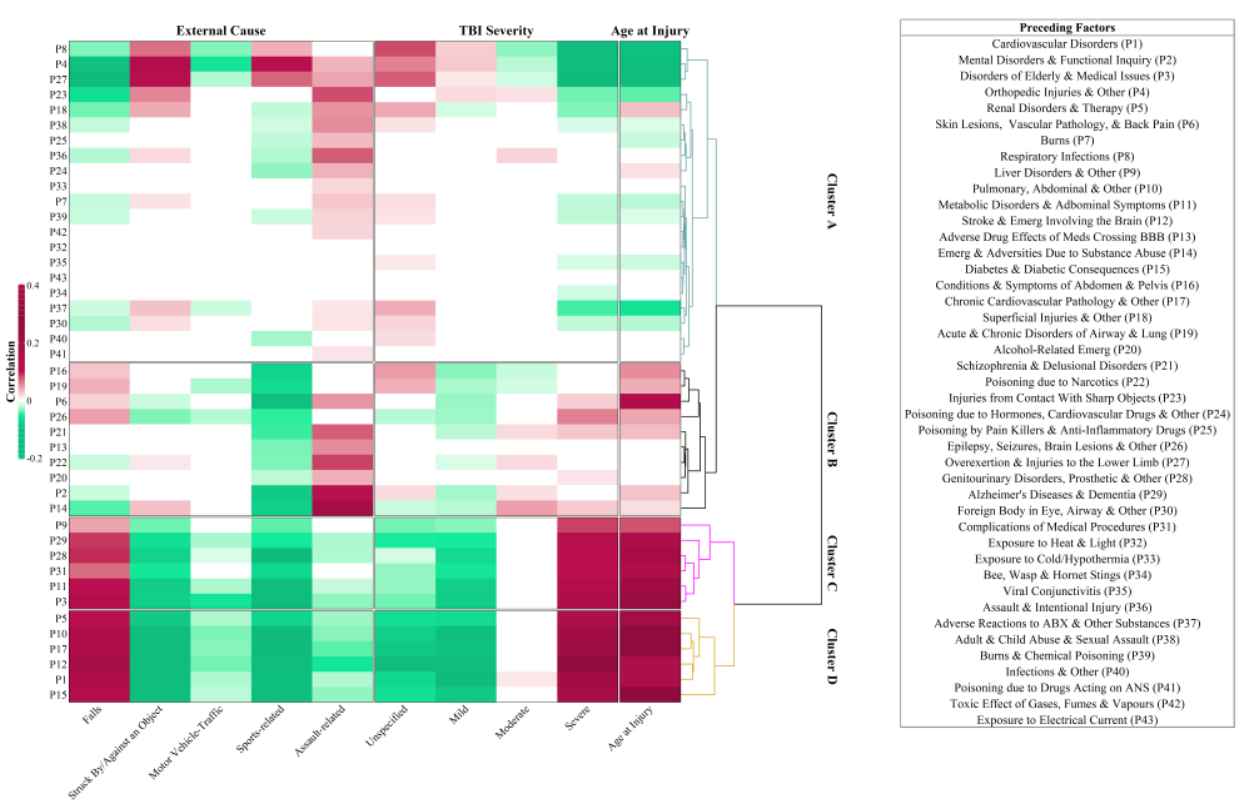


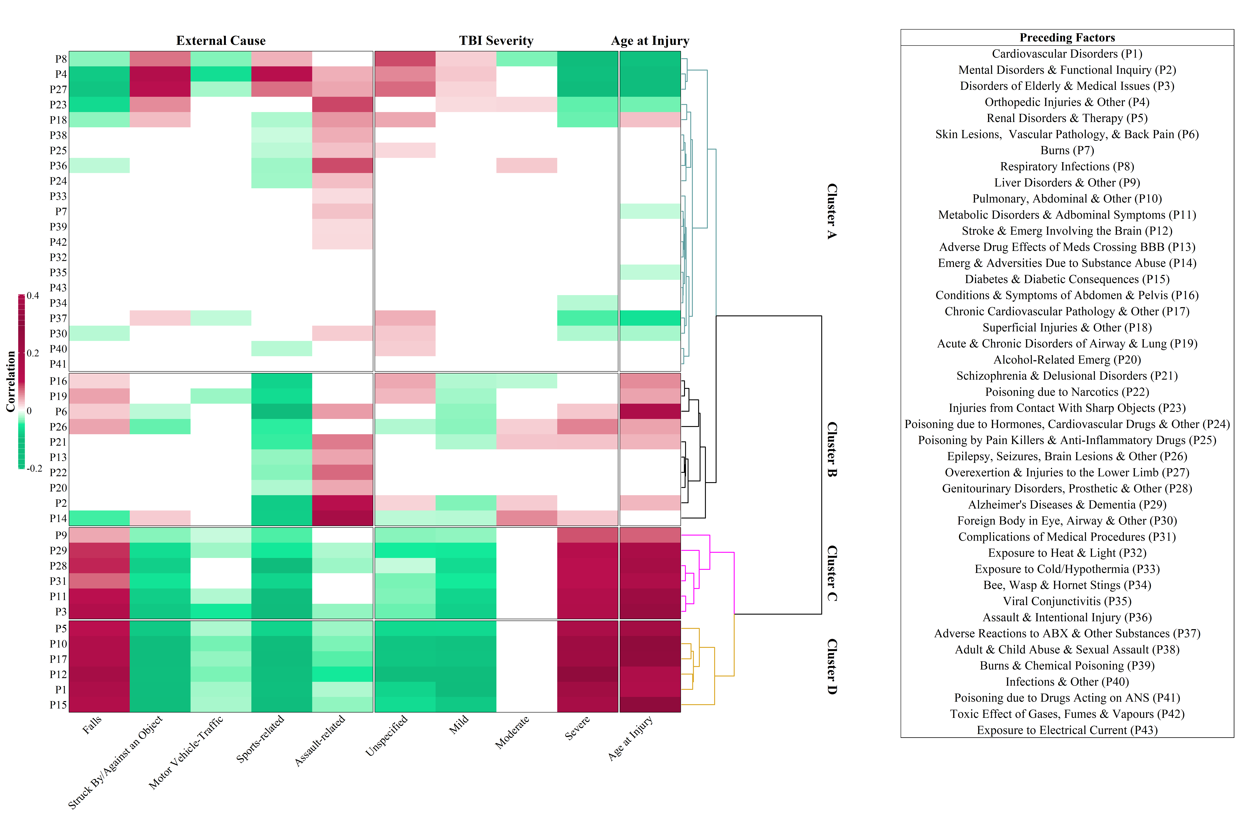


# **Supplementary Figure 4: Schematic presentation of the study design.** Health status pattern recognition (cluster analysis) before the index date and associated injury events’ patterns.


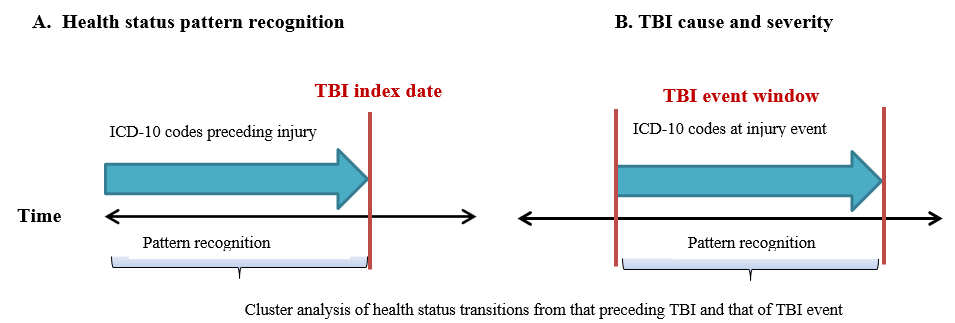

Supplement: Supplementary file 1 — Supplementary Information. [file 41598_2022_8782_MOESM1_ESM.docx]
